# Functional Interaction between HEXIM and Hedgehog Signaling during *Drosophila* Wing Development

**DOI:** 10.1371/journal.pone.0155438

**Published:** 2016-05-13

**Authors:** Duy Nguyen, Olivier Fayol, Nicolas Buisine, Pierrette Lecorre, Patricia Uguen

**Affiliations:** 1 UMR-S1174, Univ. Paris-Sud, Inserm, Université Paris-Saclay, Bât. 440, 91405 Orsay, France; 2 MNHN, UMR CNRS 5166, 75231 Paris, France; University of Massachusetts Medical School, UNITED STATES

## Abstract

Studying the dynamic of gene regulatory networks is essential in order to understand the specific signals and factors that govern cell proliferation and differentiation during development. This also has direct implication in human health and cancer biology. The general transcriptional elongation regulator P-TEFb regulates the transcriptional status of many developmental genes. Its biological activity is controlled by an inhibitory complex composed of HEXIM and the 7SK snRNA. Here, we examine the function of HEXIM during *Drosophila* development. Our key finding is that HEXIM affects the Hedgehog signaling pathway. HEXIM knockdown flies display strong phenotypes and organ failures. In the wing imaginal disc, HEXIM knockdown initially induces ectopic expression of Hedgehog (Hh) and its transcriptional effector Cubitus interuptus (Ci). In turn, deregulated Hedgehog signaling provokes apoptosis, which is continuously compensated by apoptosis-induced cell proliferation. Thus, the HEXIM knockdown mutant phenotype does not result from the apoptotic ablation of imaginal disc; but rather from the failure of dividing cells to commit to a proper developmental program due to Hedgehog signaling defects. Furthermore, we show that *ci* is a genetic suppressor of *hexim*. Thus, HEXIM ensures the integrity of Hedgehog signaling in wing imaginal disc, by a yet unknown mechanism. To our knowledge, this is the first time that the physiological function of HEXIM has been addressed in such details *in vivo*.

## Introduction

Transcription of protein-coding genes is mediated by RNA polymerase II (RNA Pol II) whose processivity is tightly controlled by the positive transcription elongation factor b (P-TEFb) after transcriptional initiation [1,2]. This kinase promotes productive transcription elongation by catalyzing the phosphorylation of a number of regulatory factors, namely the Negative elongation factor (NELF), the DRB-sensitivity inducing factor (DSIF), as well as the C-terminal domain (CTD) of RNA Pol II [3].

In human cells, P-TEFb forms two alternative complexes, which differ in size, components, and enzymatic activity [2,4]. A “small complex” (SC), composed of CyclinT and CDK9, corresponds to the catalytically active P-TEFb. In contrast, P-TEFb is kept in a catalytically inactive state and forms a “large complex” (LC) when bound by a macromolecular complex containing the 7SK snRNA, Bicoid-interacting protein 3 (BCDIN3), La-related protein 7 (LARP7), and Hexamethylene bis-acetamide inducible protein 1 (HEXIM1). The formation of the LC is reversible and P-TEFb can switch back and forth between LC and SC in a very dynamic manner. Thus, HEXIM, together with other factors, acts as a sink of active P-TEFb which regulates its biological availability at target genes [5] in response to the transcriptional demand of the cell [6–9]. Although HEXIM target genes are not known, many lines of evidence strongly support a connection between developmental pathways or diseases and the control of transcription by HEXIM [10].

Transcriptional pause was initially described in the late 80s for the *Drosophila* HSP90 gene, where transcription stalls shortly after the elongation start and RNA Pol II accumulates at the 5' end of the gene, which is thus poised for transcription [11]. It has been proposed that this phenomenon may be more general, as virtually all developmental genes in *Drosophila* [12,13] and approximately 20 to 30 percent of genes in human and mouse show similar properties [14,15]. The release from pause and the transition to productive elongation is under the control of the NELF factor [16], and so to P-TEFb, which is in turn controlled by HEXIM. Given that these genes already completed transcriptional initiation and that mRNA synthesis started, release from pause allows for a very fast and synchronized transcriptional response with low transcriptional noise.

It has been proposed that sustained pause may be a potent mechanism to actually repress gene transcription. This leads to the apparent paradox where transcriptional repression requires transcriptional initiation (reviewed by [17]). Therefore, knockdown of the transcriptional pausing factor HEXIM would release transcription and reveal the regulation of poised genes.

HEXIM1 has been initially identified as a 359 aa protein whose expression is induced in human vascular smooth muscle cells (VSMCs) following treatment with hexamethylene bis-acetamide (HMBA) which is a differentiating agent [18]. It is also called estrogen down-regulated gene 1 (EDG1) due to its decreased expression by estrogen in breast cancer cells [19,20]. Ortholog of HEXIM1 in mice and chickens is activated in heart tissue during early embryogenesis, and was so named cardiac lineage protein 1 (CLP-1) [21,22]. HEXIM1 is involved in many kinds of cancer, viral transcription of HIV-1, cardiac hypertrophy, and inflammation [10]. Overall, HEXIM defects are strongly associated with imbalance in the control of proliferation and differentiation. The CLP-1/HEXIM1 null mutation is embryonic lethal in mice, and results in early cardiac hypertrophy. Heterozygous littermates are still affected but with a less severe phenotype and survived up to adulthood [22–25]. Moreover, Mutation in the carboxy-terminal domain of HEXIM1 causes severe defects during heart and vascular development by reducing the expression of vascular endothelial growth factor (VEGF), which is essential for myocardial proliferation and survival [26–28]. Overexpression of HEXIM1 in breast epithelial cells and mammary gland decreases estrogen-driven VEGF expression, whereas it is strongly increased in loss of function mutant. As reported recently, HEXIM1 expression is required for enhancing the response to tamoxifen treatment in breast cancer patients [29]. In addition, increased HEXIM1 expression correlates with a better prognosis and decreases probability of breast cancer recurrence [20,29,30]. Additionally, terminal differentiation of murine erythroleukemia cells induced by HMBA or DMSO correlates with elevated levels of both HEXIM1 mRNA and protein. Furthermore, in neuroblastoma cells, HEXIM1 overexpression inhibits cell proliferation and promotes differentiation [31,32]. Moreover, HEXIM1 modulates the transcription rate of NF-κB, an important regulator of apoptosis, cell proliferation, differentiation, and inflammation [33]. However, despite theses advances, the dissection of HEXIM functions was mostly approached on a biochemical basis, and to date, very little is known about its physiological and developmental relevance in an integrated model. In order to address this important point, we developed an *in vivo* model and recently showed that a similar P-TEFb regulation pathway also exists in *Drosophila*, and that HEXIM is essential for proper development [34].

In *Drosophila*, the Hedgehog (Hh) signaling pathway controls cell proliferation, differentiation and embryo patterning [35–40]. The Hh activity is transduced to a single transcription factor, Cubitus interruptus (Ci) [38,41–44], the *Drosophila* homolog of Gli [37,39]. Wing imaginal discs can be subdivided into two compartments based on the presence of Hh protein. The posterior compartment (P) expresses Engrailed (En), which activates Hh and represses *ci* expression. The anterior compartment (A) expresses *ci*. The full length Ci protein (called Ci^155^) is constitutively cleaved into a truncated protein acting as a transcriptional repressor (Ci^75^) of *hh* and Decapentaplegic (*dpp*) genes. Hh inhibits the proteolytic cleavage of Ci, which then acts as a transcriptional activator of a number of target genes (Patched (*ptc*) and *dpp*, to name a few) [45]. Thus, Ci^155^ is accumulated at the boundary between the A and P compartments where there are high levels of Hh, and it is absent in P compartment [46]. Ci regulates the expression of Hh target genes in a manner dependent on Hh levels [47]. In addition to proteolytic cleavage, the biological activity of Ci is also modulated by phosphorylation and nucleo-cytoplasmic partitioning [48–51]. The mis-regulation of any components of the Hh pathway usually modifies the Ci^155^ levels, and results in developmental defects [52,53].

In this paper, we examine the function of HEXIM during *Drosophila* development. We show that HEXIM knockdown disrupts organ formation. In the wing disc, this latter effect is mediated by a strong ectopic induction of Hh signaling followed by apoptosis. The death of proliferative cells is subsequently compensated by proliferation of the neighboring cells: this is the mechanism of apoptosis-induced cell proliferation (see [54]). Ci, the transcriptional effector of Hh pathway, is highly accumulated at both mRNA and protein levels in cells where HEXIM is knocked-down. Thus, the severe phenotype of HEXIM mutants resulted from Hh-related wing patterning defects. Furthermore, we also show that *ci* acts as a genetic suppressor of *hexim*, suggesting that HEXIM is an interacting factor of the Hh signaling pathway. To our knowledge, this is the first time that the physiological function of HEXIM has been addressed in a whole organism.

## Materials and Methods

### Fly stocks and transgenes

All stocks were maintained and raised under standard conditions. Fly crosses were performed at 29°C, unless otherwise indicated. For any phenotype, at least 80 progenies from 2 to 4 independent crosses were analysed, unless specified otherwise. The phenotype penetrance was 100%, unless specified otherwise. Two RNAi-mediated *Hexim* knockdown mutants were used, one on chromosome II, the other on III (ID transformants 34633 and 34632, respectively, Vienna *Drosophila* Resource Center). To date, the stock 34633 is not available anymore from VDRC. The specificity of this RNAi-mediated knockdown has been confirmed, as previously described [34], by rescuing HEXIM knockdown phenotypes with a UAS-HEXIM strain over-expressing HEXIM. The RNAi *Hexim* probe sequence targets both *Drosophila Hexim* mRNA isoforms [34]. Fly strains were obtained from different sources, as follows: *UAS-Hex* (home-made transgene, BestGene), *UAS-hid* (gift of Hyung Don Ryoo), *UAS-p35* (gift of Jean-Philippe Parvi), *UAS-dMyc*, *UAS-CycE*, *UAS-Ci-RNAi*, *UAS-Tub-RNAi*, *UAS-Act-RNAi*, *sca-Gal4*, *GMR-Gal4*, *so-Gal4*, *ey-Gal4*, *rn-Gal4*, *Canton S*, *Dpp-lacZ*, *UAS-Hh-RNAi* (number 31475) (Bloomington stock center), *Hh-lacZ* (gift of Thomas Kornberg), *Sp/Cyo;MKRS/TM6*, *Sp-Cyo/SM5-TM6* (gift of Jean-Philippe Parvi). Additional strains were constructed by association approach: *UAS-p35;rn-Gal4*, *UAS-p35;UAS-Hex-RNAi*, *UAS-Hex-RNAi;Hh-lacZ*, *UAS-Hex-RNAi;Dpp-lacZ*, *UAS-Hex-RNAi;UAS-Ci-RNAi*, *Dpp-lacZ;rn-Gal4*, *UAS-Hex-RNAi;UAS-Hex*, *UAS-Hex-RNAi;UAS-Hh-RNAi*.

### Immunocytochemistry

For antibody staining, imaginal discs of third-instar larvae were dissected in PBS 1X, and fixed with fixation buffer (4% formaldehyde in PBS 1X) for 20 mins at room temperature. The discs were then blocked in blocking solution (PBS 1X, 0.1% BSA, 0.3% Triton X-100) for at least 1h, before being incubated with the primary antibody overnight at 4°C. We used the following primary antibodies: mouse anti-GFP (1:2000, Sigma), rabbit anti-Caspase 3 (1:200, Cell Signaling), mouse anti-Delta (1:50, DSHB), rat anti-Serrate (1:50, gift of Irvine), mouse anti-Notch intra- and extra-cellular (1:100, DSHB), mouse anti-Patched (1:50, DSHB), mouse anti-Wingless (1:500, DSHB), rat anti-Sal (1:300, gift of John F. de Celis), mouse anti-Cut (1:100, DSHB), mouse anti-Engrailed (1:50, DSHB), rat anti-Ci (1:50, DSHB), rabbit anti-P-H3 (1:200, Millipore), mouse anti-beta galactosidase (1:1000, Promega), rabbit anti-beta galactosidase (1:2000, gift from A. Plessis). The rabbit anti-dHEXIM (1:2000) was custom made by Genecust. After incubation with primary antibodies, the discs were washed four times with blocking buffer, and incubated with the appropriate fluorescent secondary antibody diluted in blocking buffer (1:200) for at least 2h at room temperature. The following secondary antibodies were used: anti rabbit, mouse, rat, and sheep antibodies (Alexa 488, 568, and Jackson Immunoresearch Cy3, Cy5). Finally, the discs were washed several times with washing buffer, and mounted in Glycerol 80%. Images were captured by Nikon Eclipse confocal microscopy and processed by Adobe Photoshop CS4 software. Immunocytochemistry was carried out on 5 to 10 independent wing discs and a representative disc is shown. In all experiments using double RNAi-mediated knockdown mutants, an immunocytochemistry of both knockdown proteins (*i*.*e*. HEXIM and Ci, or HEXIM and Ptc) were performed to control the RNAi efficiency.

### EdU Click-iT^TM^ cell proliferation assay

The EdU assay was performed using the Click-iT^TM^ cell proliferation kit (Invitrogen) following instructions. Briefly, third-instar wing discs were dissected and incubated in 10μM EdU incubation buffer for 1h. The wing discs were then fixed in fixation buffer and permeabilized by 0.5% Triton-X 100 for 20 mins, before washing several times in washing buffer (PBS 1X, 3% BSA). Discs were then incubated in 200μl of Click-iT reaction cocktails in humid chamber, at room temperature for 30 mins. Finally, discs were rinsed in washing buffer and processed to nuclear localization by DNA staining (DAPI, Roche) after mounting on slides.

### TUNEL cell death assay

TUNEL assay was carried out using the *in situ* cell death detection kit (Roche) following protocol of the producer with minor modifications. Briefly, wing discs of third-instar larvae were dissected, fixed, and permeabilized with fixation buffer (PBS 1X, Triton-X 100, PIPES, EDTA, 4% formaldehyde) in 20 mins. Fixed discs were then washed several times with washing buffer (PBS 1X, Triton-X 100), before incubated with TUNEL reaction mixture containing TdT enzyme and fluorescein-dUTP. The incubation process was performed in humid chamber, at 37°C for at least 2h. The enzymatic reaction that added the fluorescein-dUTP to free 3’-OH groups of broken DNAs was subsequently stopped by washing 4 times with washing buffer. Discs were finally mounted on slides in Glycerol 80%, and captured by fluorescent microscopy.

### X-galactosidase staining

Wing discs were dissected, and fixed with fixation buffer (PBS 1X, MgCl_2_, 0.5% glutaraldehyde). Fixed discs were then washed 4 times with PBS 1X, and incubated with coloration solution (PBS 1X, 0.2% X-Gal, Ferri-ferrocyanide, MgCl_2_) for at least 2h at 37xxC, in dark chamber. The coloration reaction was stopped by washing several times with PBS 1X. Discs were finally mounted in Glycerol 80% for image processing.

### Measure of wing discs and adult wings sizes

All flies were reared in similar growth conditions, and wing imaginal discs of corresponding third-instar larvae were analyzed. The images of wing discs were taken with a 10X objective lens after fixation with 4% formaldehyde in PBS 1X for 20 mins. The wing disc size was quantified by the Histogram function in Adobe Photoshop CS4, as previously described [55]. Adult wings were dehydrated in ethanol and mounted before being imaged on a Leica MZ APO with a 10X objective lens. The relative area of the adult wing was manually outlined and determined using ImageJ software. Statistical significance was evaluated through a two-tailed, unpaired Student’s t-test.

### Quantitative real time PCR (RT-qPCR)

Specific primers used to amplify *ci* gene are the followings, SensCi1: CTTGTTGTGCATATGCGGCG, Asensci1: GGATACTCGCAAGTGTATGG. Total RNA were extracted from 80 wing discs using RNEasy kit (QiaGen), and cDNA were synthesized using Superscript II and random hexamer (Invitrogen) following supplier instructions. qPCR were performed on a CHROMO 4 instrument (Biorad), according to manufacturer’s instructions. Data were normalized over EIFgamma internal control and results were analysed according to the 2^-ΔΔCT^ method [56]. The statistical significance was evaluated through a two-tailed, unpaired Student’s t-test.

### Microarray analysis

Total RNAs were extracted from entire heads (~200 heads/sample) of wild type (*W*^*118*^) and mutant (*GMR-Gal4>UAS-Hex-RNAi*) flies with the Qiagen Tissue-lyser and the RNEasy kits. RNAs were hybridized on Affymetrix *Drosophila*_2 3' end microarrays at IGBMC (Illkirch, France). Hybridizations were carried out with four biological replicates. Technical procedures were performed following the manufacturer's instructions. Data processing was carried out within the R environment. Signal was normalized with GCRMA and differential analysis was performed with the LIMA package [57,58]. The data are available on GEO as GSE54590.

## Results

We previously showed that HEXIM knockdown leads to a variety of developmental defects [34]. Despite many attempts, we failed to generate loss-of-function alleles of *Hexim*. Since no deficiency stock exists in *Hexim* region, we addressed the function of HEXIM during development using RNAi-mediated gene knockdown coupled to the UAS-Gal4 enhancer trap system [59], in the proliferative and differentiating regions of imaginal discs.

### HEXIM knockdown induces cell death and transient systemic proliferation arrest

**Fig 1:**
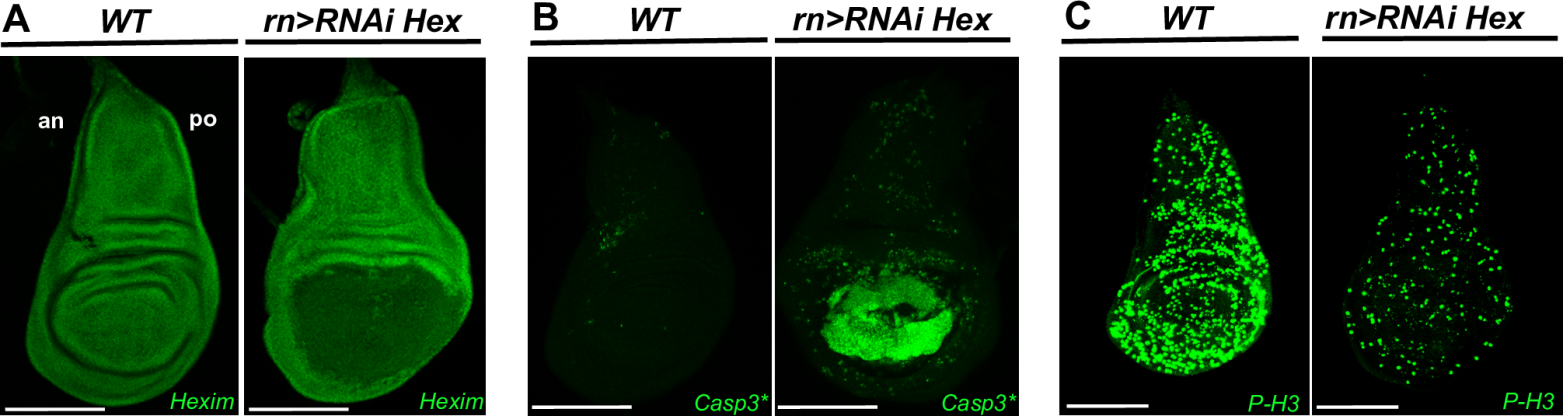
HEXIM knockdown induces cell death and transient systemic proliferation arrest. (A) Expression of HEXIM in WT and *rn-Gal4>RNAi Hexim* wing discs at early L3 stage. (B) Immunodetection of cleaved caspase 3 (Casp3*) and (C) Phospho-Histone 3 (P-H3) in WT and *rn-Gal4>RNAi Hexim* wing discs at early L3 stage. The scale bar is for 100μm. In this and all subsequent figures, wing discs are orientated anterior (an) at left and posterior (po) at right.

As previously reported, RNAi-mediated HEXIM knockdown targeted to the proliferative region of wing disc severely impedes the development of the corresponding region [34] (Fig 1A). We thus suspected that these developmental failures can result either from the death of cells in imaginal disc or a deregulation of developmental signaling pathways.

We first addressed whether these developmental defects were mediated by apoptosis by following the activity of caspase 3, the mammalian ortholog of the effector caspase DriCE, in the wing pouch disc, using *rotund* GAL4 driver on RNAi *Hexim* mutants (*rn>RNAi Hex*) (Fig 1B). As expected, the levels of developmentally programmed activated caspase 3 were extremely low in the wild type (WT) discs. In contrast, they were strongly induced in the wing pouch of the HEXIM knockdown flies (Fig 1B, *rn>RNAi Hex*). Levels of activated caspase 3 increased progressively during larval growth (S1A Fig). The latter was delayed by three to four days. Cell death was further confirmed by TUNEL assay (S2 Fig). Thus we can suggest there is a direct or indirect connection between HEXIM knockdown and apoptosis.

We next tested whether these developmental failures were due to proliferation defects by motoring the entry in mitosis and S-phase with phospho-histone 3 (P-H3) labeling and EdU incorporation assay, respectively. As expected, WT discs showed high levels of P-H3 positive cells (Fig 1C) and strong incorporation of EdU (S3A Fig). In contrast, in *rn>RNAi Hex* mutant, these markers showed poor labeling not only in the wing pouch, but also in the entire wing disc (Fig 1C and S3A Fig, *rn>RNAi Hex*), where HEXIM expression is not affected by RNAi-mediated expression (Fig 1A). This proliferation arrest is detected at both early and late L3 stages (S3B Fig). Such transient and non-autonomous reduction of proliferation is known to occur in damaged tissues after induction of apoptosis [60,61].

**Fig 2:**
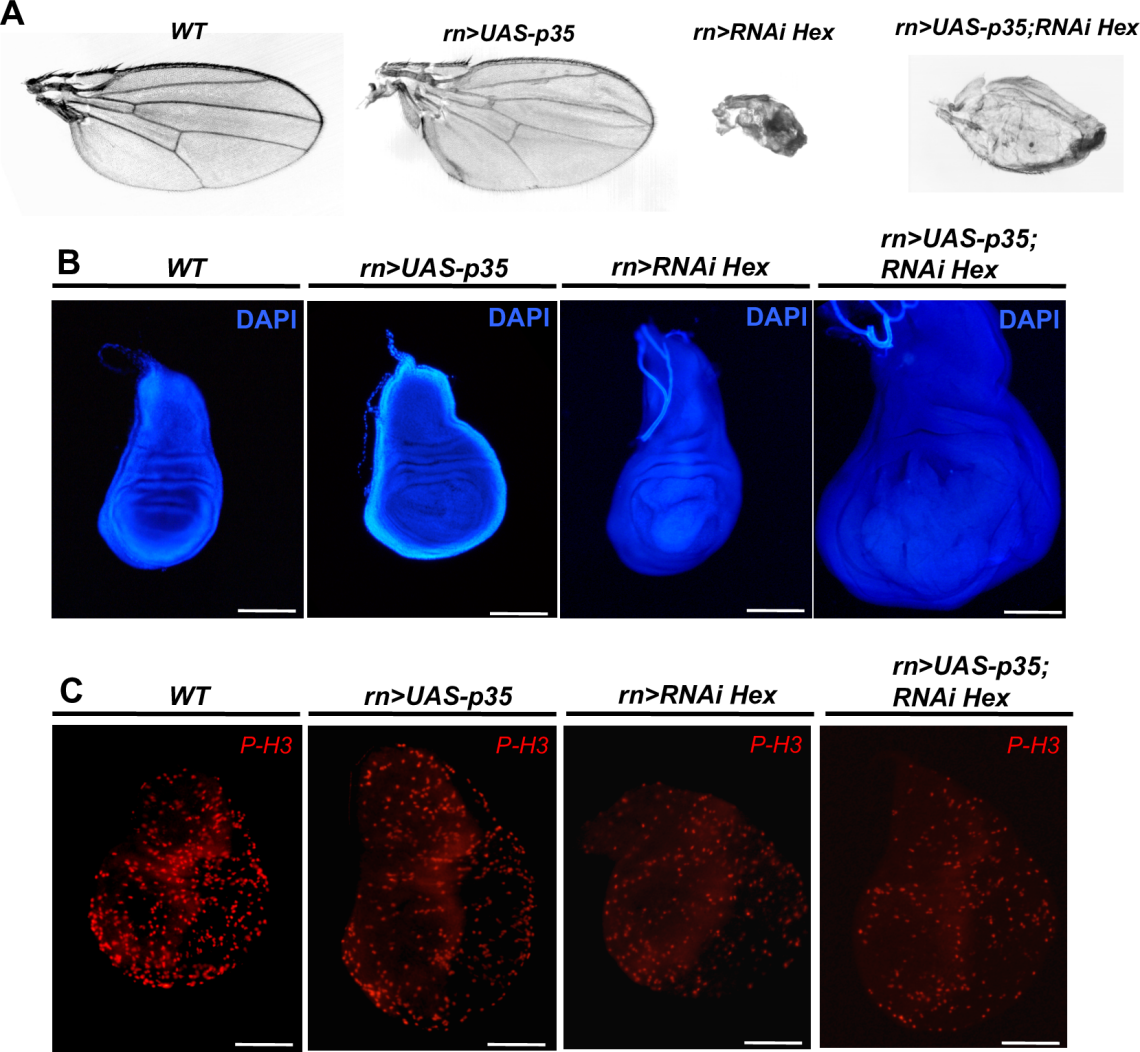
Co-expression of p35 or dMyc/Cyclin E partially rescue HEXIM knockdown phenotype. (A) Adult wing, (B) DAPI stained wing disc and (C) Phospho-Histone 3 (P-H3) immunodetection at early L3 stage of WT, *rn-Gal4>UAS-p35*, *rn-Gal4>RNAi Hexim* and *rn-Gal4>UAS-p35*; *RNAi Hexim* flies.

If the defects of the HEXIM knockdown were only limited to an induction of apoptosis and/or a reduction of the cell proliferation rate, one would expect to rescue this phenotype by co-expression with either the p35 inhibitor of caspase 3 [62,63] or enhancers of cell proliferation, such as CycE or dMyc [64]. In fact, co-expression of p35 failed to rescue the HEXIM knockdown phenotype. Of note, the corresponding flies usually died at pupal stage, except for a few escapers (10.4% of the progeny) harboring a partial rescue (Fig 2A, *rn-Gal4>UAS-p35*; *rn-Gal4>RNAi Hex*). Co-expression of CycE or dMyc rescued the wing phenotype for 57.5% of the progeny with CycE and for 70.7% with dMyc.

Taken together, these experiments show that HEXIM knockdown results in a progressive induction of apoptosis and transient proliferation arrest. In order to characterize further the relative impact of proliferation and apoptosis on the HEXIM knockdown phenotypes, we addressed whether wing developmental pathways were affected.

### HEXIM knockdown triggers apoptosis-induced compensatory proliferation and affects cell fate commitment

Despite early proliferation arrest in HEXIM mutant, the mutant wing disc was bigger than the WT one by the end of larval growth (Fig 2B, *WT* and *rn>RNAi Hex*). The latter is delayed by an average of three to four days. At early L3 stage, the number of P-H3 positive cells is only half that of WT flies (Fig 2C, *WT* and *rn>RNAi Hex*). But after one to two more days, the proliferation rate increases before dropping down at the end of the delayed time (S3B Fig, *WT* and *rn>RNAi Hex*). The increased wing size, together with delayed larval growth, phenocopies the compensatory proliferation induced by apoptosis in the wing pouch [55,60,65]. Although the global proliferation rate in HEXIM mutant is lower than the WT, it is compensated by a longer growth time, finally leading to larger wing discs (x1.8-fold) (Fig 2B and S3C Fig, *WT* and *rn>RNAi Hex*). Furthermore, in HEXIM knockdown flies co-expressing p35, the wing disc size is even larger (2.6-fold higher; Fig 2B and S3C Fig, *rn>UAS-p35; RNAi Hex*), due to many “undead cells” continuously producing mitogenic signals [55,66,67]. Of note, overexpression of p35 in WT background induces a slight overgrowth of wing disc (x1.4-fold; Fig 2B, *rn>UAS-p35*) due to inhibition of developmental apoptosis, as previously described [67].

We next sought to identify the signaling pathways that are impaired during wing disc development. We first examined the expression patterns of the Notch pathway’s components. We showed that HEXIM knockdown effectively abrogated the expression of Delta (Dl) and Serrate (Ser) in the Ventral (V) and Dorsal (D) compartments, although expression is maintained in the stripe between the D-V boundary (S4C and S4D Fig). In addition, the expression of Notch (N) intra- and extra-cellular components was completely repressed in the wing pouch (S4I and S4J Fig). The expression levels of other markers (Sal, Cut, and En) follow a similar trend (S4F and S4H Fig). Therefore, HEXIM knockdown strongly affects wing patterning. In addition, we also examined the expression levels of Ptc and the two morphogens Dpp and Wingless (Wg), which are induced in response to tissue damage and promote compensatory proliferation [55,60–61,65,67–68]. In third instar WT wing discs, Wg is expressed along the D-V boundary as well as in two concentric circles at the border and outside of the wing pouch [65] (S4E Fig). In contrast, Dpp and Ptc are expressed in the A compartment, restricted at the A-P boundary, in which Dpp expression is further extended by a 7-cell-diameter region along the A-P stripe, compared to Ptc (S4A and S4B Fig). In HEXIM knockdown mutants, the expression of both Dpp and Ptc was increased (S4A and S4B Fig). Of note, the expression domain of Dpp was also enlarged in the A-P stripe. Moreover, we found that HEXIM knockdown resulted in high accumulation of Wg in the cells surrounding the wing pouch, along with a weak, extended stripe at the D-V boundary (S4E Fig). Thus, the expression patterns of Wg, Dpp and Ptc markers in HEXIM mutant wing discs resemble the patterns observed when apoptotic cells promote compensatory proliferation [65,68–69]. Taken together, our results show that HEXIM knockdown affects cell fate commitment of the proliferative cells of the wing imaginal disc.

### HEXIM knockdown deregulates Hh signaling and induces Ci expression

**Fig 3:**
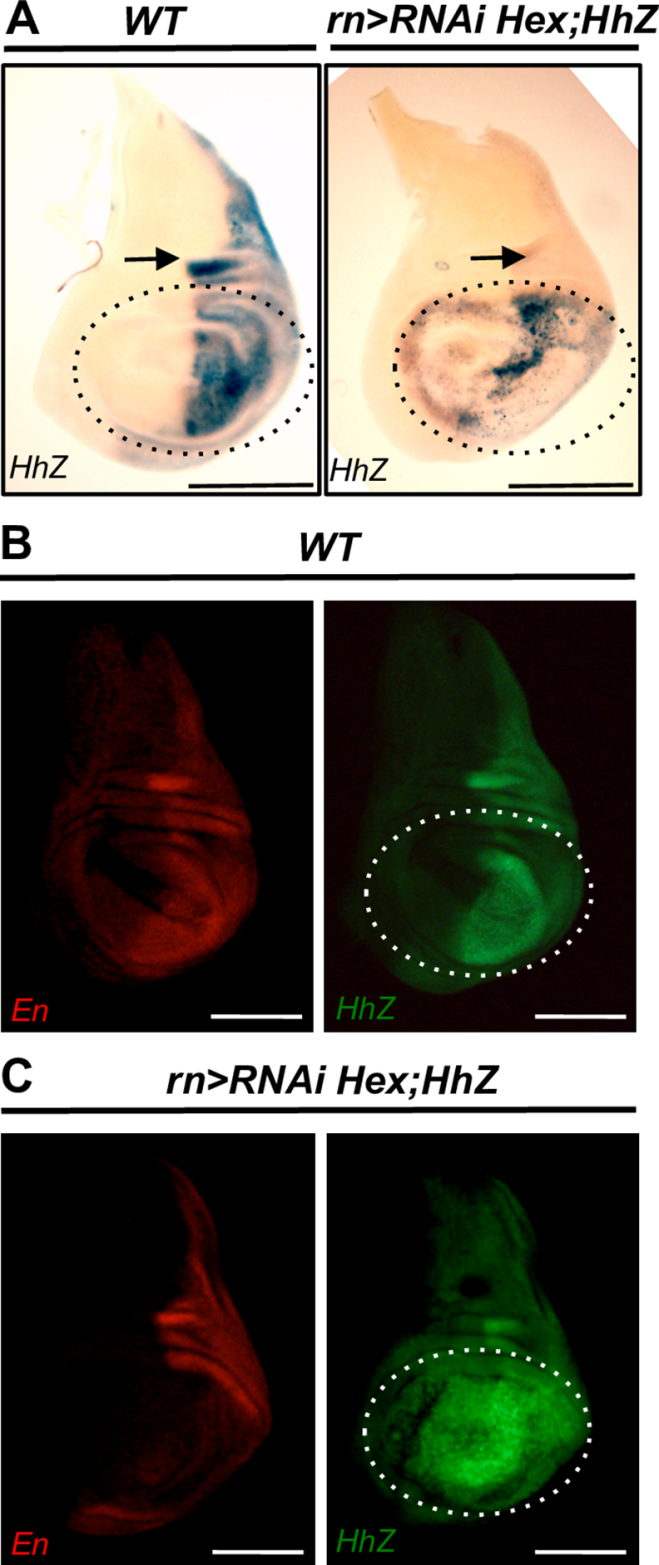
HEXIM knockdown deregulates Hh signaling pathway. (A) X-Gal staining of *hh-lacZ* reporter, in WT and *rn-Gal4>RNAi Hexim* wing discs. For the mutant, β-galactosidase staining duration was reduced to limit signal saturation. En and Hh-LacZ β-galactosidase co-immunodectection in WT (B) and in *rn-Gal4>RNAi Hexim* (C) wing discs. Arrows indicate the non-autonomous down-regulation of Hh in the posterior compartment of the notum part. The wing pouch is marked with dotted white line. The assays were performed at early L3 stage.

To precisely characterize Hedgehog signaling, we monitored *hh* expression in HEXIM knockdown flies with a *hh-lacZ* reporter. Strikingly, we found a profoundly altered *hh* expression, which was expressed not only in the canonical anterior part but also in the posterior part of the wing pouch (Fig 3A–3C). It is noteworthy that co-labeling of Hh-LacZ and En (a posterior marker of wing disc) reveals aberrant definition of anterior-posterior territories in HEXIM knockdown imaginal disc (Fig 3B–3C). Importantly, proliferative cells typically do not respond to apoptosis signal by increasing Hh expression, as it is the case for differentiated cells [54]. Therefore, the ectopic expression of Hh in HEXIM knockdown mutants is not a simple response to the ongoing apoptosis of the wing disc. We also note that Hh levels drop down non-autonomously in the notum part of the P compartment (Fig 3A, arrow), which is surprising because the notum is properly developed in the HEXIM mutant. This reduced level of Hh may be transient.

**Fig 4:**
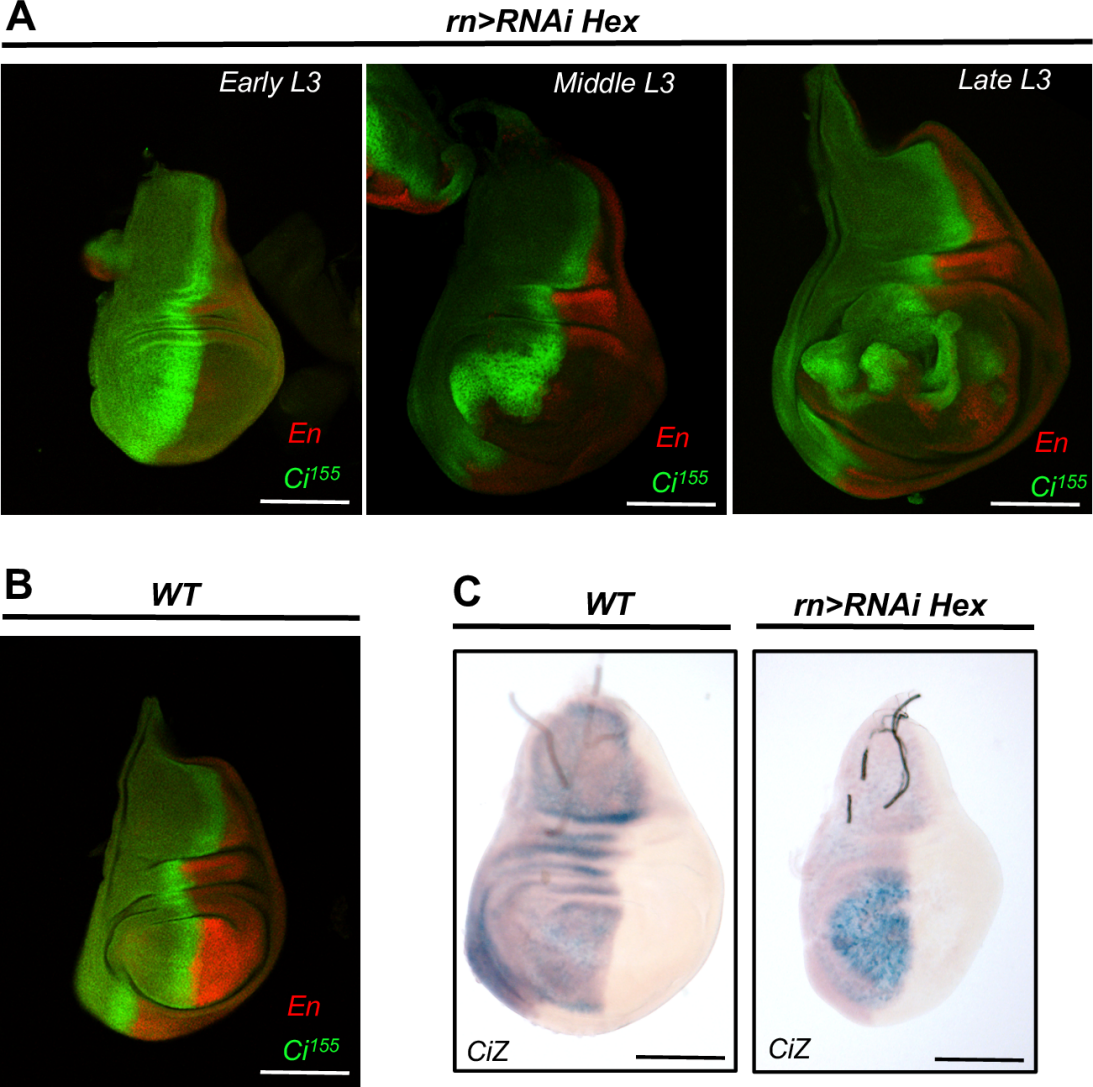
HEXIM knockdown deregulates Ci^155^ expression at both protein and transcript levels. Expression of Ci^155^ and En in *rn-Gal4>RNAi Hexim* (A) and WT (B) wing discs, at various L3 stages. (C) Transcription of the *Ci-lacZ* reporter in WT and *rn-Gal4>RNAi Hexim* wing discs.

We next characterized the timing and expression profile of Ci, the transcriptional effector of the Hh pathway [38,41–44]. In HEXIM mutants, we found a broader expression domain of Ci^155^ that expanded beyond the A-P stripe into the anterior part of the disc. This accumulation of Ci^155^, which is highest at early L3, persisted throughout the extended phase of larval growth (Fig 4A and 4B). In fact, time course analysis revealed that Ci^155^ accumulation started before the progressive induction of apoptosis, and that caspase-3-activated cells did not accumulate Ci^155^ (S1A and S1C Fig). Moreover, when apoptosis was blocked by co-expression of p35 in *rn-Gal4> RNAi Hexim* mutant, Ci^155^ protein level was still high at the A-P stripe (S5 Fig). This result shows that Hh deregulation takes place before apoptosis. In addition, X-Gal staining of a *ci-lacZ* reporter revealed an increased accumulation of *ci* transcripts localized at the anterior part of the wing pouch in HEXIM mutant (Fig 4C). *ci* mRNA quantification by RT-qPCR showed a 2-fold increase in HEXIM mutant compared to WT (S6 Fig). This indicates a transcriptional regulation link between *Ci* and HEXIM. Altogether, these data show that the altered Ci^155^ expression profile is not a simple consequence of apoptosis, but rather an early response to HEXIM knockdown, which in turn, strongly affects Hh signaling, apoptosis, compensatory proliferation and wing patterning.

**Fig 5:**
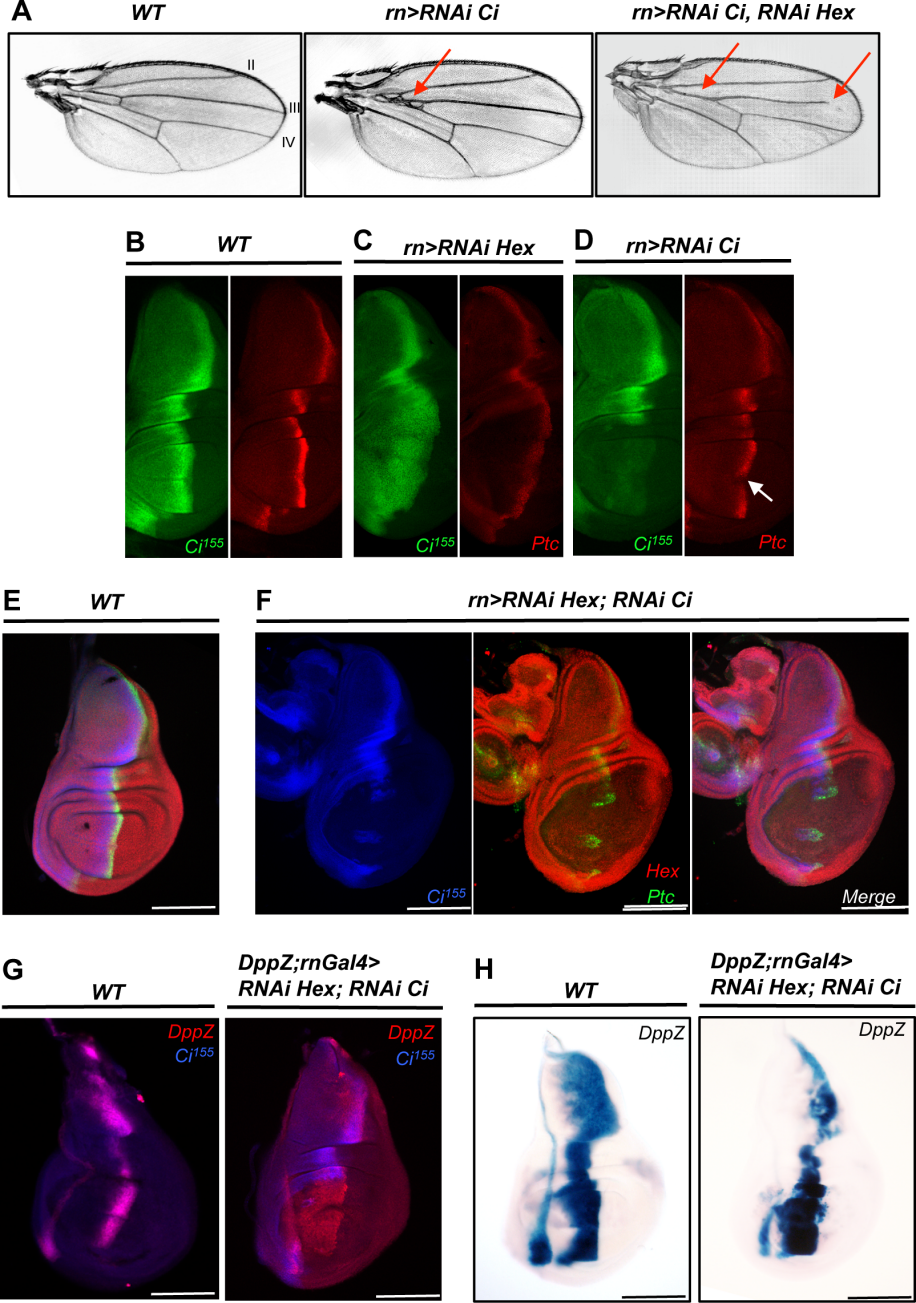
HEXIM is a regulator of Ci. (A) Wing phenotype in WT, *rn-Gal4>RNAi Ci* and *rn-Gal4>RNAi Ci; RNAi Hexim* flies. Red arrows refer to vein or intervein defects. For single or double mutants, 100% of flies displayed the phenotype (over > 200 mutants flies scored). Immuno-localization of Ci^155^ and Ptc in the wing disc of WT (B), *rn-Gal4>RNAi Hexim (C)* and *rn-Gal4>RNAi Ci* (D) flies. The reduced levels of Ci^155^ in the A-P stripe (white arrow) causes aberrations at the intervein 3 and 4. Immuno-localization of Ci^155^, Ptc and HEXIM in WT (E) and in *rn-Gal4>RNAi Hexim; RNAi Ci* double mutant (F) wing discs. (G) Immuno-localization of Ci^155^ and Dpp in WT and in *rn-Gal4>RNAi Hexim; RNAi Ci* double knockdown. (H) X-Gal staining of *dpp-lacZ* reporter in WT and in *rn-Gal4>RNAi Hexim; RNAi Ci* mutant.

**Fig 6:**
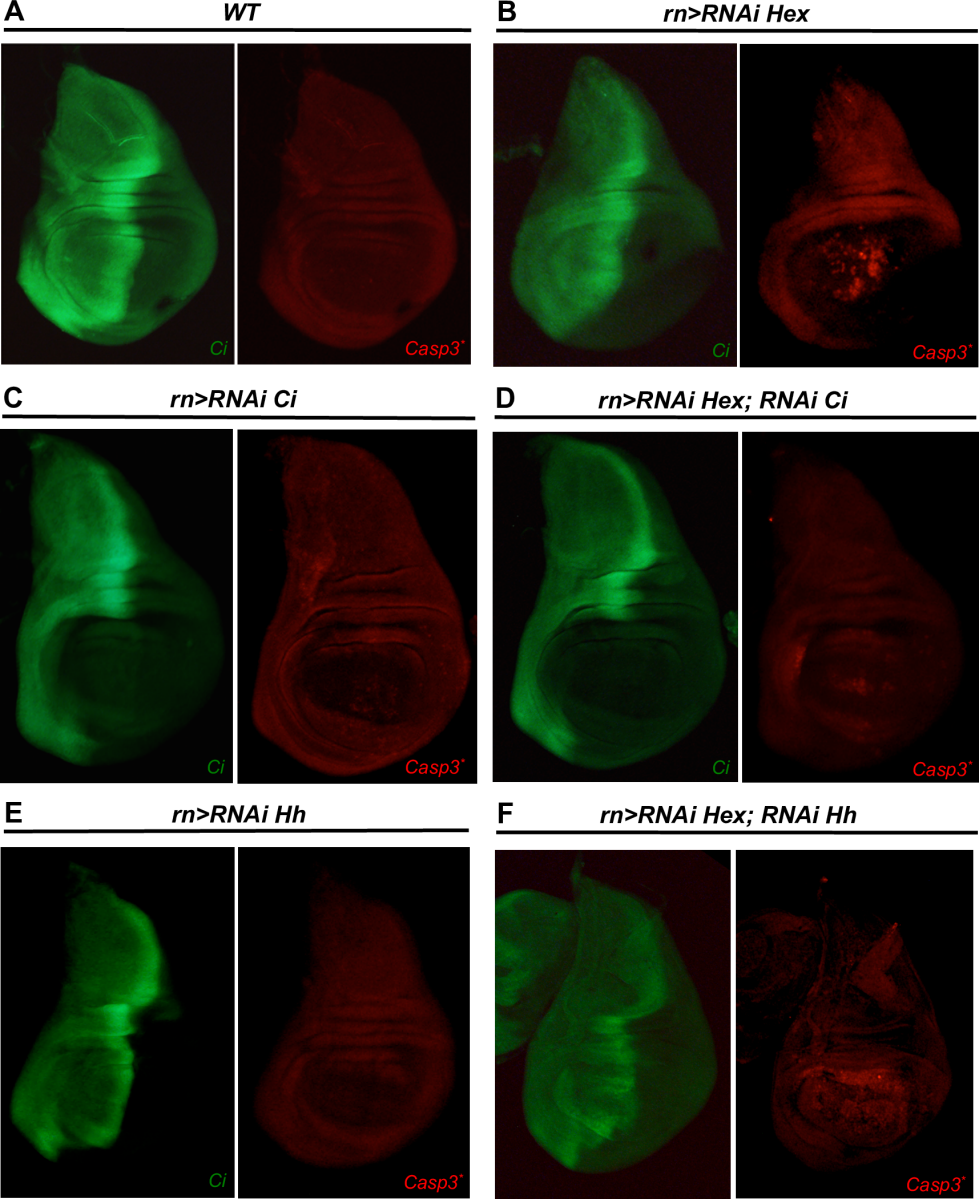
Apoptosis is reduced in double RNAi-mediated knockdown mutant of HEXIM and Ci or HEXIM and Hh. Immunodetection of cleaved caspase 3 (Casp3*) and Ci^155^ at early L3 stage of WT (A), *rn-Gal4>RNAi Hexim* (B), *rn-Gal4>RNAi Ci* (C), *rn-Gal4>RNAi Hexim; RNAi Ci* (D), *rn-Gal4>RNAi Hh* (E) and *rn-Gal4>RNAi Hexim; RNAi Hh* (F) strains.

We next probed the epistasis between HEXIM and some components of the Hh pathway. We used a combination of single and double RNAi targeting HEXIM and Ci^155^ expression in the wing pouch to monitor the expression of two known Ci^155^ target genes: *dpp* and *ptc*. In adult *rn>RNAi Ci* flies, the wing blade presented aberrations between interveins 3 and 4 (Fig 5A, *rn>RNAi Ci*), as expected [70]. The specificity and efficiency of the Ci RNAi construct were controlled by immunocytochemistry (Fig 5B–5D) and RT-qPCR, both showing a strong reduction of Ci expression (2.6-fold reduction; S6 Fig). The *rn>RNAi Ci* mutant flies display a weak but significant decrease of Ptc level in the A-P stripe, especially at the cross of the A-P and D-V boundaries (Fig 5D, *rn>RNAi Ci*, arrow), but the expression of both Dpp (data not shown) and Wg (S7C Fig, *rn>RNAi Ci*) were unaffected compared to WT (S7A Fig, *WT*). This agrees well with the known biology of the Hh pathway, in which Ptc expression requires more Ci^155^ than Dpp [71]. We then addressed the combined effect of both HEXIM and Ci^155^ knockdowns. Strikingly, in double mutant, the strong HEXIM phenotype was almost fully rescued since all flies developed wings similar to single Ci knockdown mutant, but with a missing anterior crossvein and an altered vein 3 (Fig 5A, *rn-Gal4>RNAi Ci; RNAi Hex*, *red arrows*). In double knockdown mutant, Ptc expression was abrogated in the wing pouch due to the absence of Ci (Fig 5, compare E, *WT* and F, *rn-Gal4>RNAi Ci; RNAi Hex*), whereas Dpp expression is expended in the A compartment probably due to the absence of Ci^75^ repressor (Fig 5G and 5H, compare *WT* and *DppZ;rn-Gal4>RNAi Hexim; RNAi Ci*). Note that other markers of wing development like Wg and Cut displayed a WT expression profile in this double mutant (S7A and S7D Fig). Furthermore, only a modest activation of caspase 3 was detected in the wing pouch of the double mutant (Fig 6A–6D), which is similar to single RNAi-mediated Ci mutant (Fig 6C). Therefore, this result suggests that *ci* is a genetic suppressor of *hexim* during wing development.

**Fig 7:**
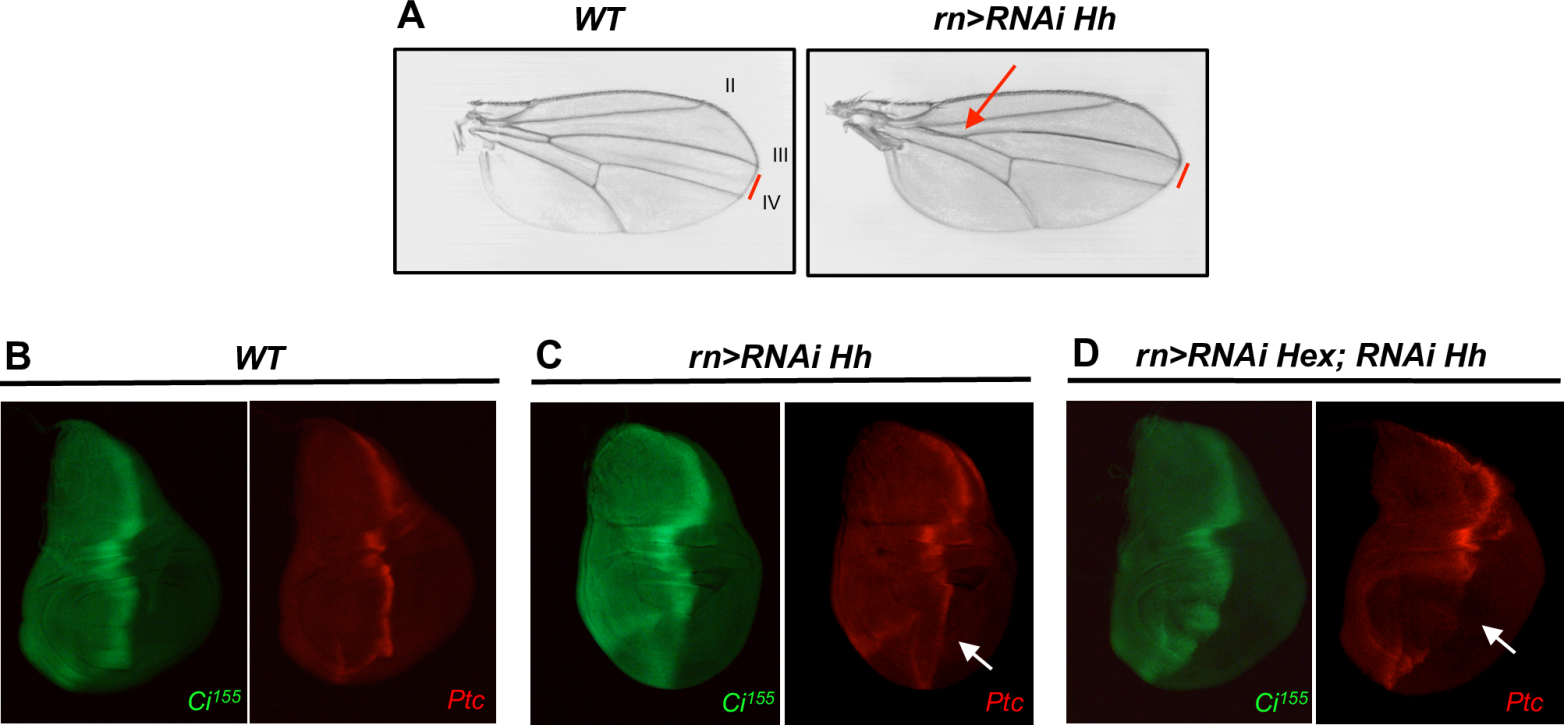
Reduction of Hh does not rescue HEXIM knockdown mutant. (A) Wing phenotype in WT and *rn-Gal4>RNAi Hh* flies. The distance between L3 and L4 veins are indicated with a red bar. Immuno-localization of Ci^155^ and Ptc in WT (B), *rn-Gal4>RNAi Hh* (C) and *rn-Gal4>RNAi Hexim; RNAi Hh* double mutant (C) wing discs. The reduced levels of Ptc in the A-P stripe (white arrow) are marked in single and double mutants.

We then tested whether Hh ectopic overexpression caused by HEXIM knockdown could trigger apoptosis, by using single and double RNAi against Hh and HEXIM. Single *rn>RNAi Hh* mutant displayed reduced anterior crossvein and L3-L4 intervein area (S1 Table; Fig 7A), as expected [72,73]. Consistently, wing discs show a strong decrease of Ci^155^ and Ptc immuno-staining (Fig 7B and 7C, compare *WT* with *rn>RNAi Hh* strains), with no apoptosis (Fig 6E) and WT expression profile of Wg and Cut (S7E Fig). Similarly, double *rn>RNAi Hexim; RNAi Hh* mutants also display reduction of Ci^155^ and Ptc immuno-staining (Fig 7D), and modest levels of caspase 3 (Fig 6F) compared to single *rn>RNAi Hexim* mutant. Therefore, apoptosis activation in HEXIM mutant is indeed dependent on a high expression level of Hh. Of note, the development of *UAS-Hex-RNAi; UAS-Hh-RNAi* mutant is blocked at pupal stage. In addition, Wg and Cut expression profiles are highly affected (S7F Fig), in a manner similar to *rn>RNAi Hexim* mutant (S7B Fig). This may not be surprising because it is already known that Hh knockdown mutant may have a different phenotype from Ci mutant [74].

Altogether, our results strongly suggest that HEXIM is a functional (direct or indirect) interacting partner in the Hedgehog signaling pathway.

### HEXIM knockdown similarly affects other differentiating and proliferating tissues

**Fig 8:**
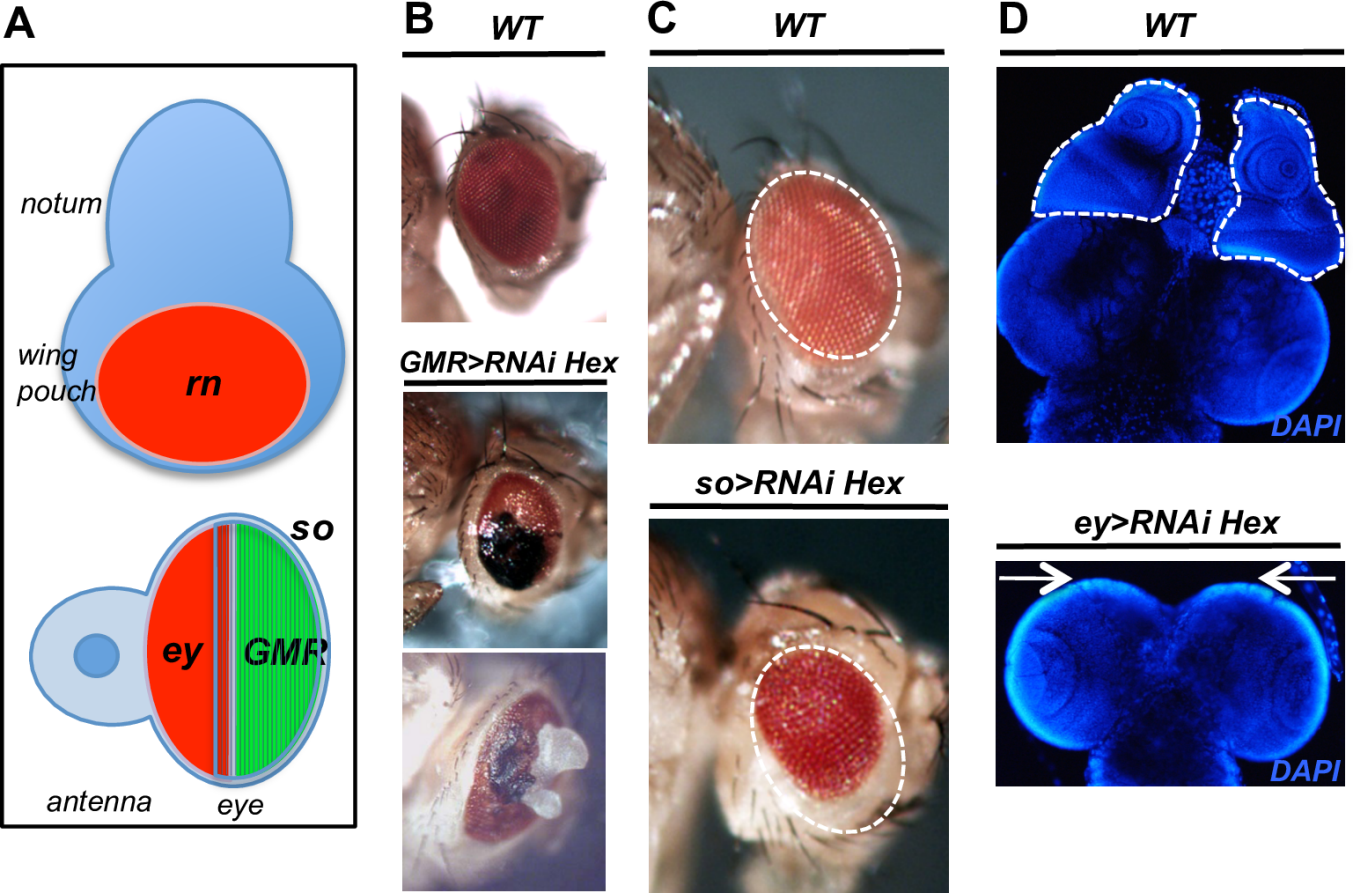
HEXIM knockdown affects both differentiating and proliferating tissues. (A) Schematic diagram of the eye-antenna disc summarizing the expression patterns of the *Gal4* drivers: *ey* (red), *GMR* (green) and *so* (hatched). (B) WT and *GMR-Gal4>RNAi Hexim* eyes. (C) WT and *so-Gal4>RNAi Hexim* eyes. (D) WT and the *ey-Gal4*>*RNAi Hexim e*ye-antenna imaginal discs (white circles) and brain. Note the absence of eye-antenna discs in *ey-Gal4*>*RNAi Hexim* (white arrows).

We next addressed whether other differentiating or proliferating tissues respond in a similar manner to HEXIM knockdown. To this end, we focused on different parts of the eye imaginal disc. When targeted to the differentiating cells located in the posterior part of the eye discs, HEXIM knockdown resulted in a mild phenotype with rough and/or black-spotted eyes of smaller size in adult flies (*glass multiple reporter*, *GMR>RNAi Hex*), without noticeable alteration of the eye disc morphology [34] (Fig 8A and 8B). In some rare cases (4 over 983 flies), a mass of overgrowing cells protrudes through the eyes (Fig 8B) suggesting a deregulation of cell proliferation in this mutant. In contrast, when targeted to the anterior (proliferative) part of the eye discs with the *eyeless* driver (*ey>RNAi Hex*), the phenotype was much more pronounced and resulted in the ablation of the entire eye-antenna discs (Fig 8A and 8D). The corresponding headless larvae died before hatching. Moreover, HEXIM knockdown driven by *sine oculis* GAL4 driver *(so-Gal4)*, which follows an increasing gradient from the anterior to the posterior compartment of the eye discs [75] (Fig 8A), resulted in flies with an intermediate phenotype of rough and smaller eyes (25 to 40% smaller than WT; Fig 8C and S1 Table). These data show that in the eye disc, HEXIM knockdown affects both proliferating and differentiating cells, at different degrees. Furthermore, HEXIM knockdown leads to apoptosis in the eye imaginal disc, as observed in the wing disc. Indeed, in *GMR>RNAi Hex* mutant activated caspase 3 is significantly accumulated in the differentiating region, posterior to the secondary mitotic wave (S8 Fig).

In order to further characterize the molecular phenotype of HEXIM knockdown in differentiating cells, we conducted Affymetrix microarrays gene expression analysis of the *GMR>RNAi Hex* adult eyes (Fig 8B). We found approximately 200 genes differentially expressed, in which one-half were up-regulated while the other half were down-regulated (S2 Table). Probably owing to the necrosis in the eye territories, the expression levels of genes involved in the immune system were remarkably elevated (e.g. attacin, cecropin, semmelweis). The expression of several markers of eye terminal differentiation was reduced (e.g. rhodopsin, sepia, irregular chiasm C-roughest) in HEXIM knockdown mutants, while the expression of other genes was induced (e.g. mre11, metabolic genes). These results show defects of cell fate commitment during eye disc development in HEXIM mutants.

Therefore, HEXIM knockdown affects cell fate commitment in both proliferating and differentiating cells, although the mechanism may be cell-specific and dependent on their ongoing developmental programs.

## Discussion

### RNAi-mediated HEXIM knockdown phenotype results from failure to commit to a developmental program

Given that HEXIM is a general regulator of transcription elongation, the transcription machinery of mutant cells is eventually expected to be strongly affected that leads to cell death. One would argue that the *rn>RNAi Hex* mutant phenotype (undeveloped wing) is likely to be a simple consequence of a severe demolition of the wing pouch. However, we clearly show that the whole tissue is not ablated, although HEXIM mutant displays significant levels of apoptosis. Indeed, dying cells are efficiently replaced by new ones through apoptosis-induced proliferation (AIP) to such extent that the wing disc, including the wing pouch, increases strongly in size but still fails to promote the proper development of the wing. Thus, the phenotype is not a consequence of reduced size of the wing pouch, but rather cells fail to commit to a proper developmental program. The ectopic induction of Hh is one (among other) clear signature of abnormal development.

### HEXIM knockdown profoundly affects Hh signaling

Two lines of evidence support a functional connection between HEXIM and Hedgehog signaling: 1) Ci expression is induced early, and 2) *ci* is a genetic suppressor of *hexim*.

Althought Hh is supposed to be mainly anti-apoptotic, there are a few reports indicating that it can promote apoptosis during development. For example when Ptc is deleted, there is increasing apoptosis in hematopoietic cells [76] or Shh increases cell death in posterior limb cells [77]. In our study, the induction of Hedgehog signaling is a primary event that precedes the wave of apoptosis, in HEXIM knockdown mutants. Given that cells subject to patterning defects often undergo apoptosis, the ectopic expression of Hh is probably the molecular event that triggers apoptosis in the wing disc. Then, the subsequent AIP will produce new cells and fuel a self-reinforcing loop of Hh activation and apoptosis (since HEXIM expression is continuously repressed). Accordingly, in *rn>RNAi Hex* mutant, cells undergoing AIP survive but fail to differentiate. This is supported by previous reports where deregulation of Hedgehog signaling, through modifications of Ci expression levels, leads to developmental defects [53,78,70]. The phenotype of double knockdown mutants of Ci and HEXIM can be simply explained as following: cells lack the ability to respond to Hedgehog signaling and become blind to Hh patterning defect, thus leading to a Ci-like phenotype.

**Fig 9:**
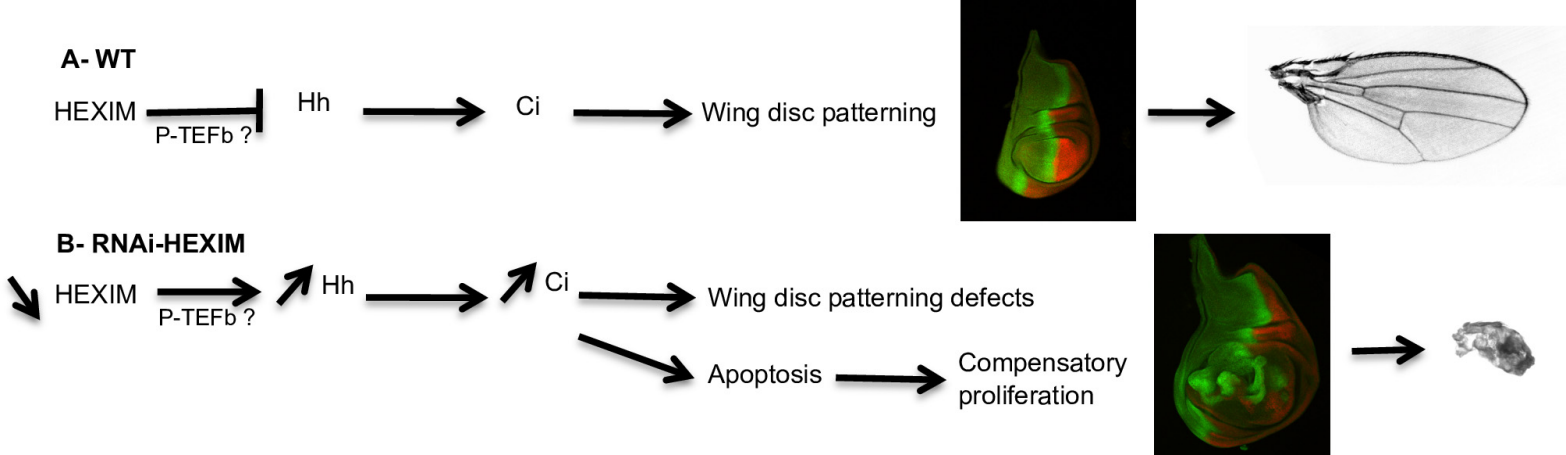
Model of HEXIM-dependent regulation of wing disc development. (A) In WT wing pouch, HEXIM regulates hedgehog signaling and so its transcriptional effector Ci. (B) In HEXIM knockdown background, Hh is strongly induced and so Ci, and provokes apoptosis, which activates apoptosis-mediated compensatory proliferation. The resulting patterning defects prevent wing development despite the compensatory proliferation.

Although we can not exclude that Ci^155^ expression is directly affected by HEXIM, the extended expression domain of Ci^155^ in *rn>RNAi Hex* mutants may also indirectly result from increased levels of Hh. Indeed, the breadth of the AP stripe is defined in part by a morphogenetic gradient of Hh, with a decreasing concentration towards the anterior part of the wing disc. Thus, the augmented levels of Hh induced in *rn>RNAi Hex* mutants could in principle explain the broader Ci expression at the AP stripe. To summarize, HEXIM knockdown increases Hh expression, potentially through regulation of P-TEFb complex [79], leading to patterning defects and a wave of apoptosis followed by compensatory proliferation (Fig 9).

A genetic screen in *Drosophila* showed that the two components of the small P-TEFb complex, Cdk9 and Cyclin T, are strong activators of the Hh pathway [79], but so far, no evidence directly connects HEXIM to Hh pathway. To this regard, our work clearly establishes this connection. It is then tempting to speculate that by knocking-down HEXIM, the levels of active P-TEFb will be eventually increased that leads to an ectopic activation of the Hh pathway. More work is needed to specifically address this mechanistic point.

Interestingly, when carried out in the eye discs, *GMR>RNAi Hex* mutants display an extreme but rare phenotype with protuberances of proliferating cells piercing through the eyes. Although we could not characterize these few events any further, the parallel with the proliferating cells, which fails to differentiate in the wing disc, is striking. Of note, the role of HEXIM in the balance between proliferation and differentiation is not quite novel (see Introduction). Indeed, HEXIM was previously reported to be up-regulated upon treatment of HMBA [18], a well known inducer of differentiation. In this paper, we show that the regulatory role of HEXIM during development is mediated via controlling the Hedgehog signaling pathway. To our knowledge, this is the first time that this has been addressed *in vivo* and in a non-pathological context.

### HEXIM: a regulator of transcription during development?

Among other functions, HEXIM acts as a regulator of the P-TEFb activity which is in turn a general regulator of elongation [34]. The availability of the P-TEFb activity mediates transcriptional pausing, a mechanism by which RNA pol II pauses shortly after transcription initiation and accumulates at the 5' end of genes. Transcription may then or may not resume, depending on a number of inputs [80]. In these cases, RNA pol II appears 'stalled' at the 5' end of genes. Release from transcriptional pausing is fast and allows a more homogeneous and synchronized transcription at the scale of an imaginal disc or organ. In the other hand, a lack of release from transcriptional pausing is also a potent way to silence transcription [81]. Interestingly, genome wide profiling of RNA pol II revealed a strong accumulation at the 5' end of 20 to 30% of the genes, most of which involved in development, cell proliferation and differentiation [12–15]. In this context, HEXIM knockdown would be expected to have strong developmental defects. We clearly see such effects in all tissues tested so far.

The patterning of WT wing disc is set by a morphogenetic gradient of Hh, with high levels in the P compartments and no expression in the A compartment. It is therefore tempting to speculate that the Hh coding gene would be in a transcriptionally paused state in the anterior part of the wing pouch, that would be released upon HEXIM knockdown. This simple molecular mechanism, although speculative, would account for the induction of the ectopic expression of Hh in the anterior part of the wing pouch and the subsequent loops of apoptosis and AIP, ultimately leading to the wing developmental defects. We tried to ask whether the distribution of RNA Pol II along *hh* and *ci* is compatible with a transcriptional pause by using a number of RNA Pol II ChIP-Seq datasets that have been generated, together with RNA-Seq data, over the past few years (e.g. [82–83]). We processed these datasets and computed the stalling index (SI) for all genes, as previously defined [15] (data not shown). The SI is computed after mapping ChIP-Seq reads on the reference genome and corresponds to the log ratio of the reads density at the 5' end of the gene over the reads density along the gene body. Although these datasets clearly reveal a number of 'stalled' genes (>>100), we could not find evidence of paused RNA Pol II for *hh* and *ci* (SI value of order 0), which were instead being transcribed. We note, however, that these datasets have been generated from whole embryos and S2 cell line. Given that Hh and Ci define morphogenetic gradients, their expression (and their transcriptional status) is likely highly variable between cells located in the different sub-regions of a disc, which may therefore not be reflected in these datasets.

### Concluding remarks

Apart from the developmental function of HEXIM that we address in this work and the connection between HEXIM and Hedgehog signaling, our results may also be of interest for human health studies. First, Hedgehog is a major signaling pathway that mediates liver organogenesis and adult liver regeneration after injury [84]. In a murine model of liver regeneration, the Hedgehog pathway promotes replication of fully differentiated (mature) hepatocytes [84]. Thus, addressing whether a connection between HEXIM and Hh exists would provide a mechanistic link between the control of gene expression and adult liver regeneration. Second, deregulated Hedgehog signaling is a common feature of many human tumors, and is found in at least 25% of cancers. In addition, recent data showed that aberrant Hedgehog signaling activates proliferation and increases resistance to apoptosis of neighboring cells and thus helps create a micro-environment favorable for tumorigenesis [85]. Since its discovery, deregulated HEXIM expression is often associated to cancers and other diseases [10]. Adding a new connection between HEXIM and Hedgehog signaling will shed more light into the role of HEXIM in abnormal development and cancer.

Surprisingly, although the biochemical interactions between HEXIM and its partners have been thoroughly described, very little is known about its biological function. Thus, this is the first time that the functional impact of HEXIM has been addressed in an integrated system.

## Supporting Information

S1 Fig Immuno-localization of activated caspase 3 and Ci^155^ in WT and during the delayed larval growth of *rn>RNAi Hexim* mutants. Expression of both Casp3* (A) and Ci^155^ (B) is described in *rn>RNAi Hexim* mutants at different stages of L3 (from early to late). A magnification of middle L3 stage shows that Ci^155^ positive cells are not apoptotic cells. Expression of En and Ci^155^ in *rn>UAS-hid* imaginal wing disc and together with Casp3* (D) is also depicted.
(TIF)

S2 Fig HEXIM knockdown induces cell death in imaginal wing discs. TUNEL assay in WT and *rn-Gal4>RNAi Hexim* wing discs at early L3 stage.
(TIF)

S3 Fig HEXIM knockdown with or without p35 leads to a transient proliferation arrest and to an increase of wing disc size. (A) EdU assay in WT and *rn-Gal4>RNAi Hexim* wing discs. (B) Number of P-H3 positive cell of WT (blue); *RNAi Hexim* (HEX^-^, green), and *UAS-p35*; *RNAi Hexim* (HEX^-^ + P35, red) wing discs during delayed larval growth. (C) Quantification of the wing disc size in WT (blue); *RNAi Hexim* (HEX^-^, green), and *UAS-p35*; *RNAi Hexim* (HEX^-^ + P35, red) strains. Counting of PH3 positive cells was performed from 5 to 10 wing discs for each genotype. Wing size is the average from 8 individuals wings. (***P<0.001; error bars: standard deviation).
(TIF)

S4 Fig Comparison between WT and *rn>RNAi Hexim* mutants of the immuno-staining of several selector genes and morphogens known to be involved in imaginal wing disc development. (C-J) Immunocytochemistry of the selector genes *Delta (Del)*, *Serrate (Ser)*, *Wingless (Wg)*, *Spalt (Sal)*, *Cut*, *Engrailed (En)*, *Notch extra-cellular (Ne)*, and *Notch intra-cellular (Ni)* in WT and *rn-Gal4>RNAi Hexim* (C’-J’) wing discs. (A,B) Immunocytochemistry of morphogens *Decapentaplegic (Dpp)* and *Patched (Ptc)* in WT and *rn-Gal4>RNAi Hexim* wing discs. The breadth of Dpp and Ptc expression are indicated with a red scale on the figures (A,B). Immunocytochemistry were performed at early L3 stage.
(TIF)

S5 Fig Patterns of Wg and Ci^155^ in *rn-Gal4>RNAi Hexim* with or without co-expression of p35. Immuno-staining at early L3 stage of Wg and Ci^155^ in WT, *rn-Gal4>RNAi Hexim*, and *rn>UAS-p35; RNAi Hexim*.
(TIF)

S6 Fig *ci* mRNA quantification by RT-qPCR in *rn-Gal4>RNAi Hexim and rn-Gal4>RNAi Ci* mutants. mRNA quantifications are means from duplicate experiments and are compared to WT condition (**P<0.01; error bars: standard deviation).
(TIF)

S7 Fig Patterns of Wg and Cut in various mutants. Immuno-staining of Wg and Cut in WT (A), *rn-Gal4>RNAi Hexim* (B), *rn-Gal4>RNAi Ci* (C), *rn-Gal4>RNAi Hexim; RNAi Ci* (D), *rn-Gal4>RNAi Hh* (E) and *rn-Gal4>RNAi Hexim; RNAi Hh* (F).
(TIF)

S8 Fig HEXIM knockdown induces cell death in eye discs. Immuno-localization at ealy L3 stage of Casp3* in WT and *GMR-Gal4>RNAi Hexim* eye discs. MF: Morphogenic Furrow. SMW: Secondary Mitotic Wave.
(TIF)

S1 Table Phenotype of Hh or HEXIM RNAi mutants. (PDF)

S2 Table List of the differentially expressed genes in the *GMR-Gal4>RNAi Hexim* mutants compared to the WT adult heads. (DOC)
