# Supplementary figures and images for: Functional Interaction between HEXIM and Hedgehog Signaling during Drosophila Wing Development
Source: PLoS One. 2016 May 13;11(5):e0155438. doi: 10.1371/journal.pone.0155438 (PMC4866710; doi:10.1371/journal.pone.0155438)

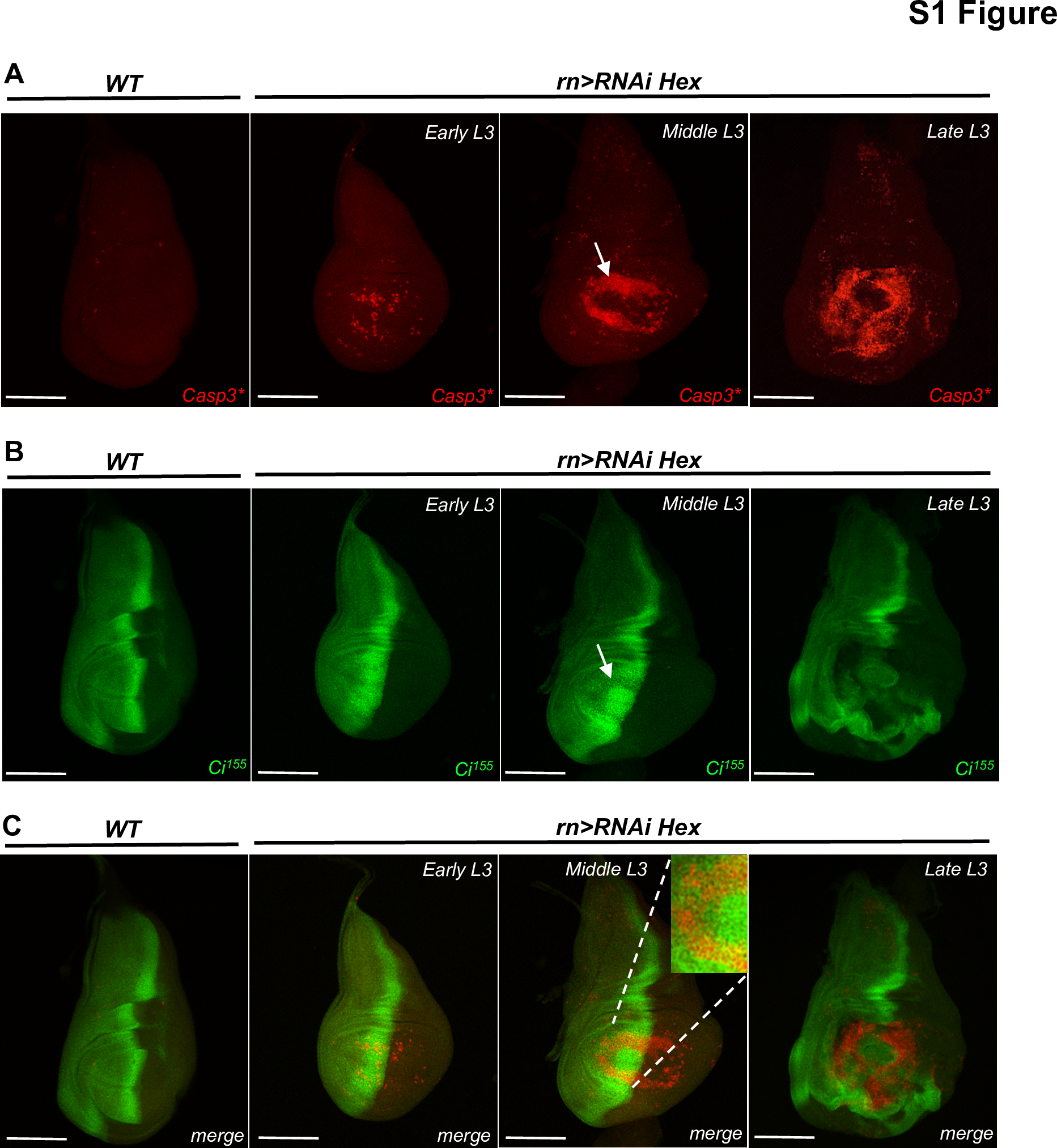

Supplement: S1 Fig — Expression of both Casp3* (A) and Ci155 (B) is described in rn>RNAi Hexim mutants at different stages of L3 (from early to late). A magnification of middle L3 stage shows that Ci155 positive cells are not apoptotic cells. Expression of En and Ci155 in rn>UAS-hid imaginal wing disc and together with Casp3* (D) is also depicted. (TIF) [file pone.0155438.s001.tif]

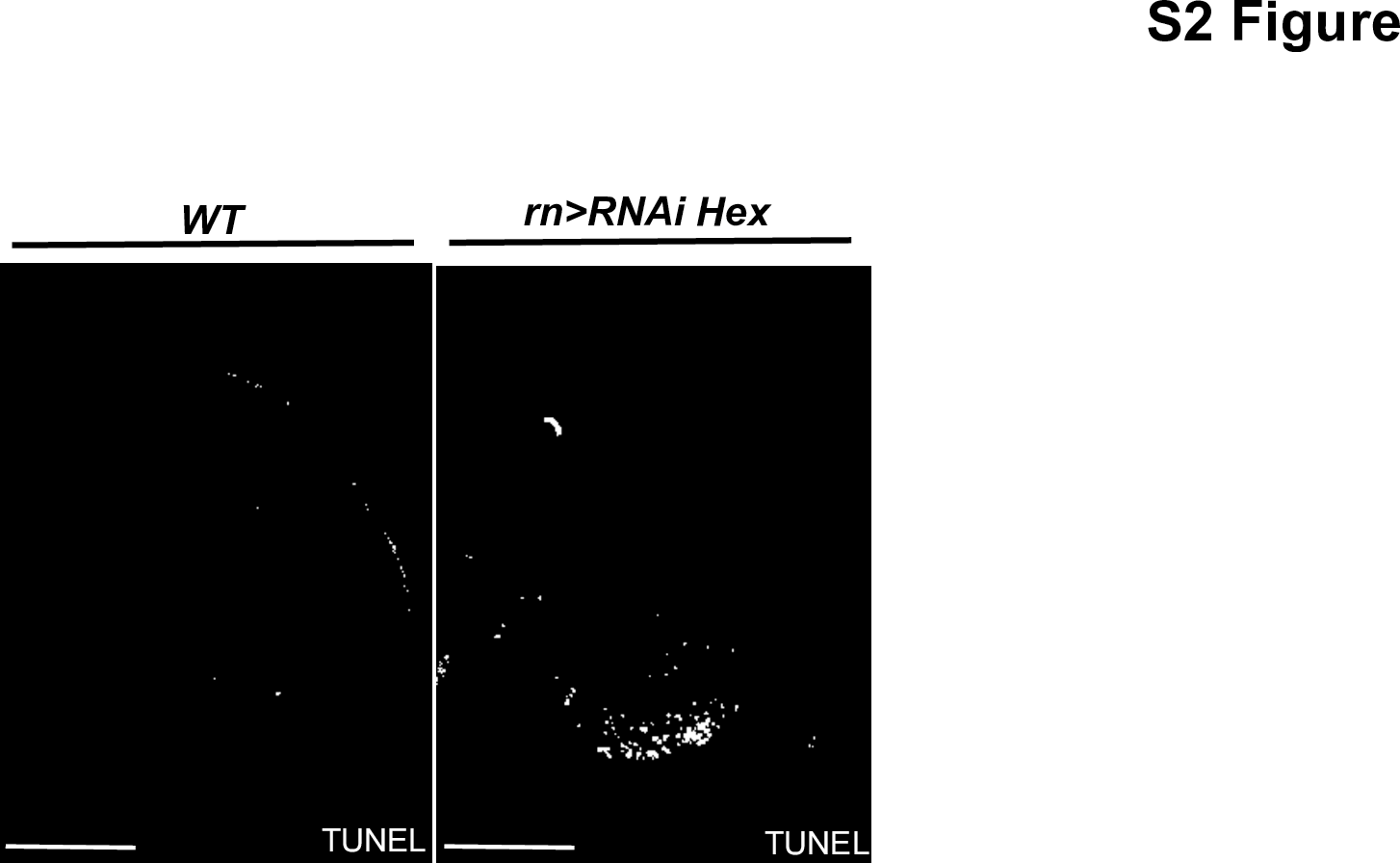

Supplement: S2 Fig — TUNEL assay in WT and rn-Gal4>RNAi Hexim wing discs at early L3 stage. (TIF) [file pone.0155438.s002.tif]

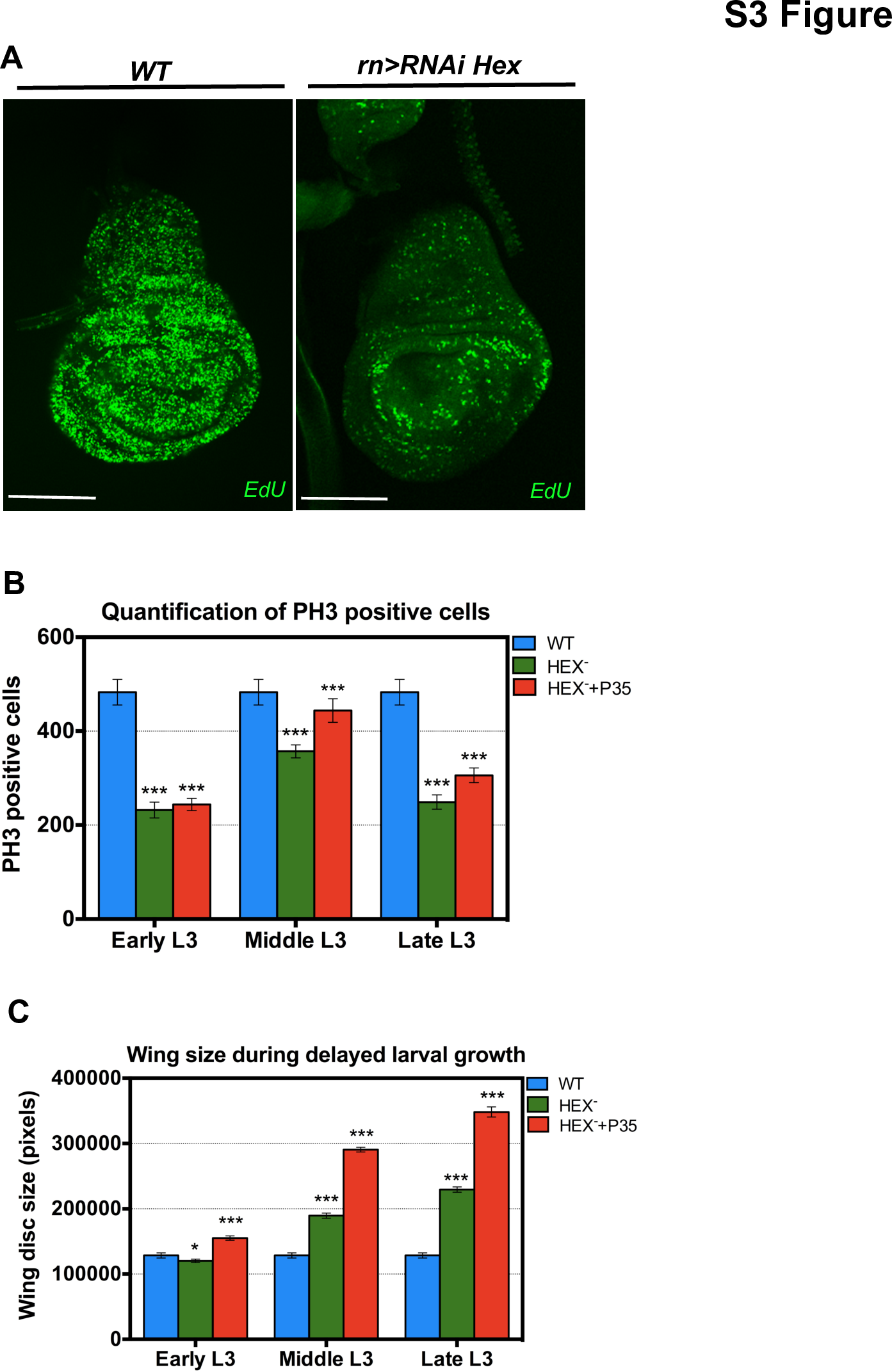

Supplement: S3 Fig — (A) EdU assay in WT and rn-Gal4>RNAi Hexim wing discs. (B) Number of P-H3 positive cell of WT (blue); RNAi Hexim (HEX-, green), and UAS-p35; RNAi Hexim (HEX- + P35, red) wing discs during delayed larval growth. (C) Quantification of the wing disc size in WT (blue); RNAi Hexim (HEX-, green), and UAS-p35; RNAi Hexim (HEX- + P35, red) strains. Counting of PH3 positive cells was performed from 5 to 10 wing discs for each genotype. Wing size is the average from 8 individuals wings. (***P<0.001; error bars: standard deviation). (TIF) [file pone.0155438.s003.tif]

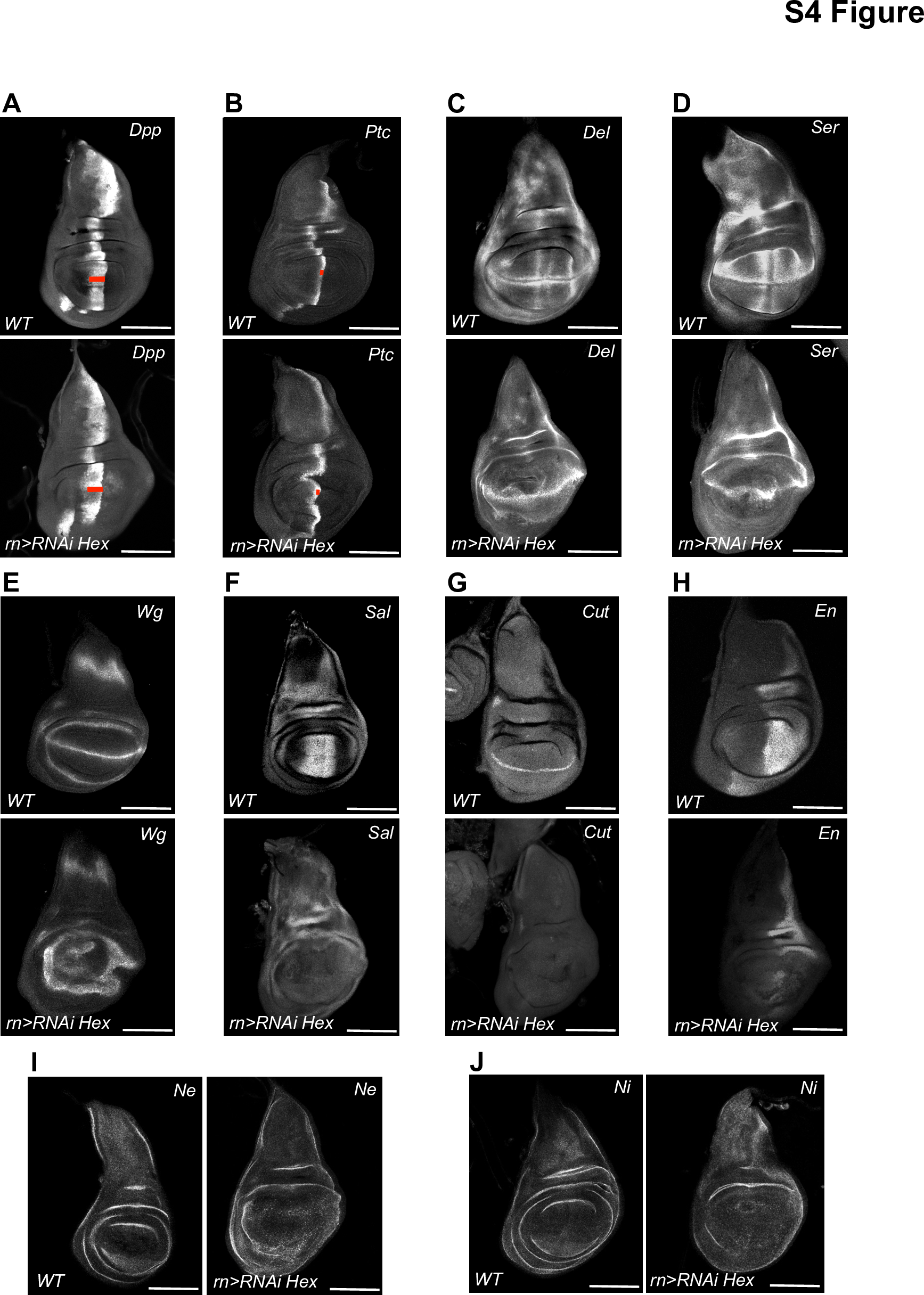

Supplement: S4 Fig — (C-J) Immunocytochemistry of the selector genes Delta (Del), Serrate (Ser), Wingless (Wg), Spalt (Sal), Cut, Engrailed (En), Notch extra-cellular (Ne), and Notch intra-cellular (Ni) in WT and rn-Gal4>RNAi Hexim (C’-J’) wing discs. (A,B) Immunocytochemistry of morphogens Decapentaplegic (Dpp) and Patched (Ptc) in WT and rn-Gal4>RNAi Hexim wing discs. The breadth of Dpp and Ptc expression are indicated with a red scale on the figures (A,B). Immunocytochemistry were performed at early L3 stage. (TIF) [file pone.0155438.s004.tif]

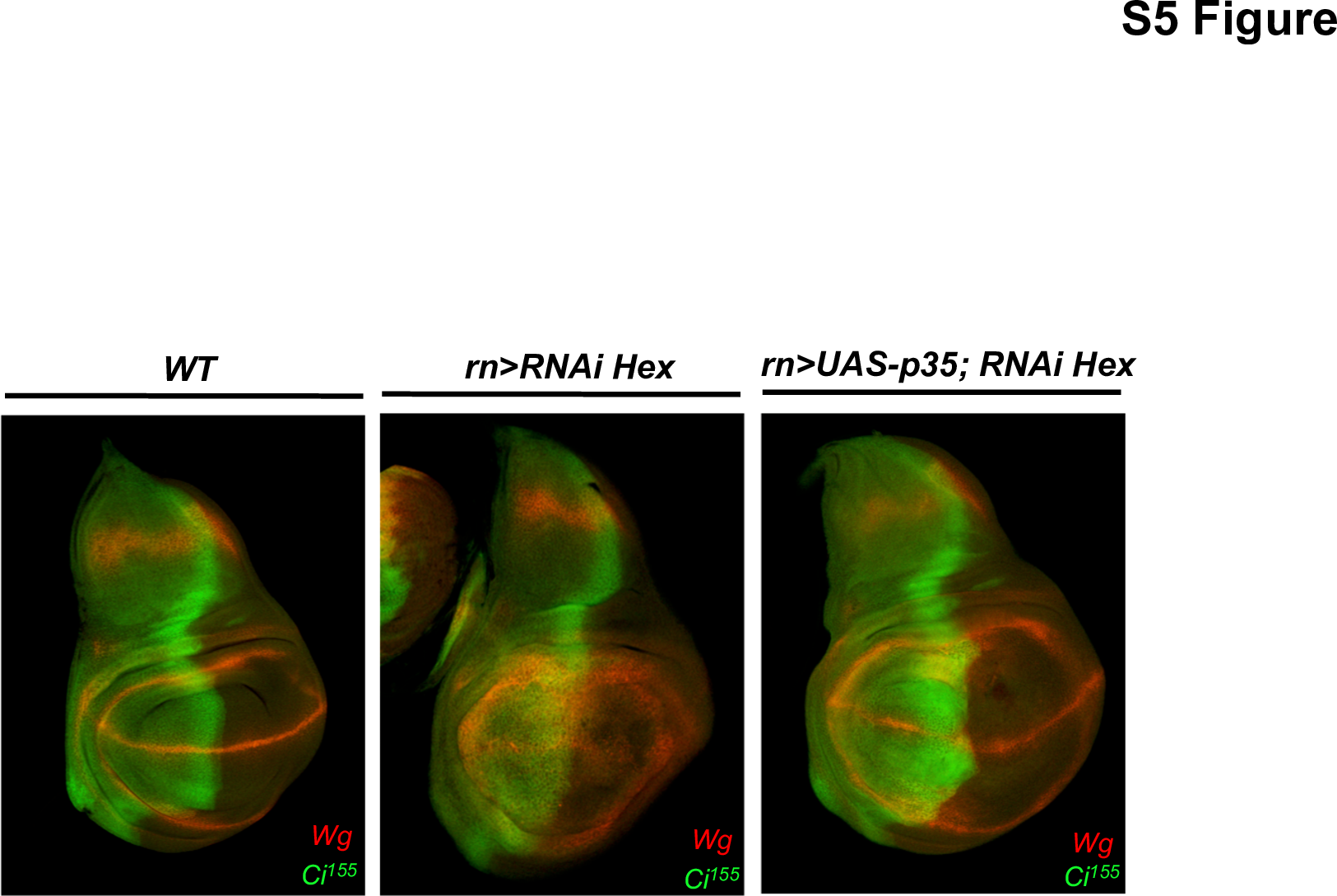

Supplement: S5 Fig — Immuno-staining at early L3 stage of Wg and Ci155 in WT, rn-Gal4>RNAi Hexim, and rn>UAS-p35; RNAi Hexim. (TIF) [file pone.0155438.s005.tif]

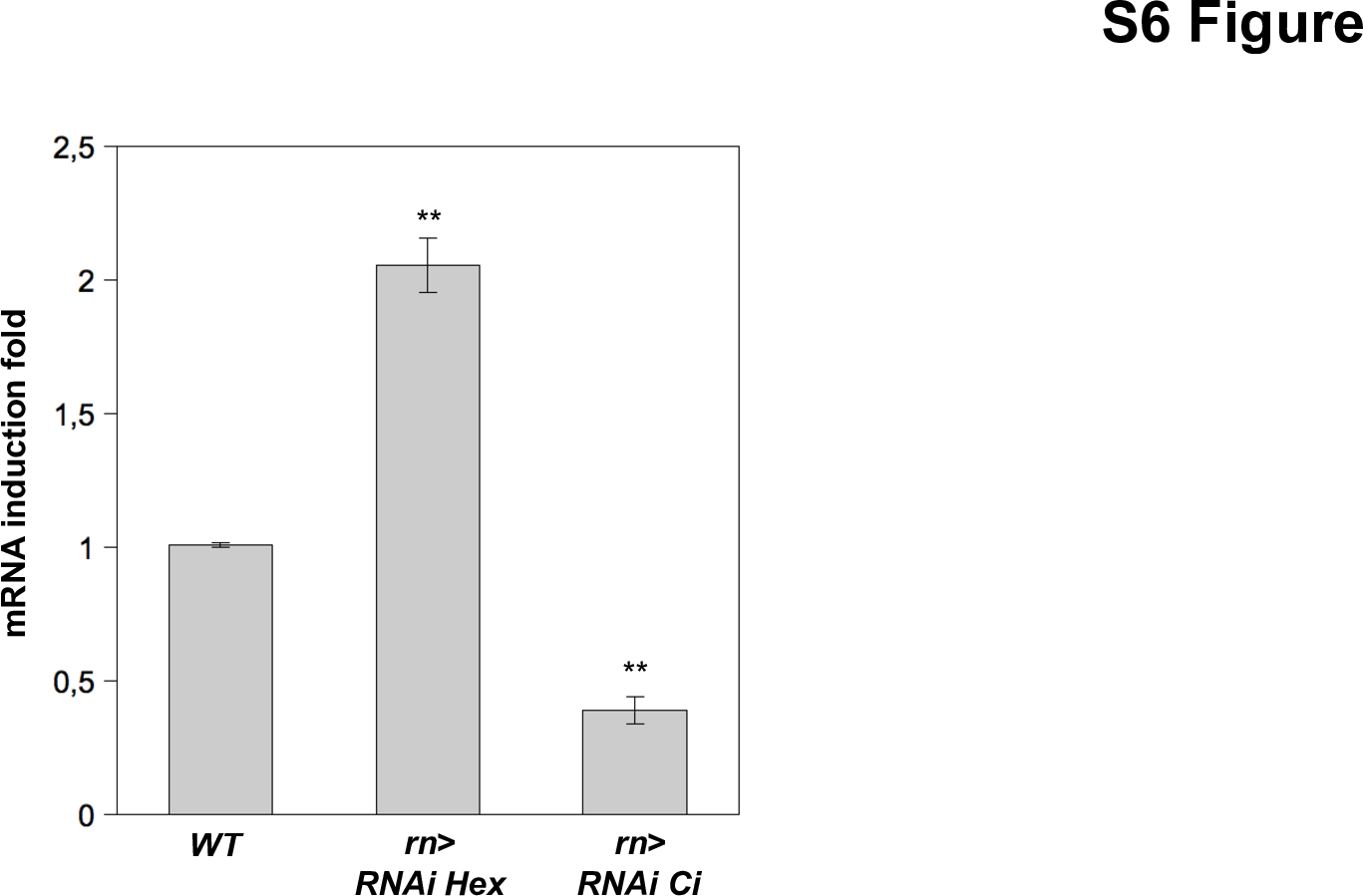

Supplement: S6 Fig — mRNA quantifications are means from duplicate experiments and are compared to WT condition (**P<0.01; error bars: standard deviation). (TIF) [file pone.0155438.s006.tif]

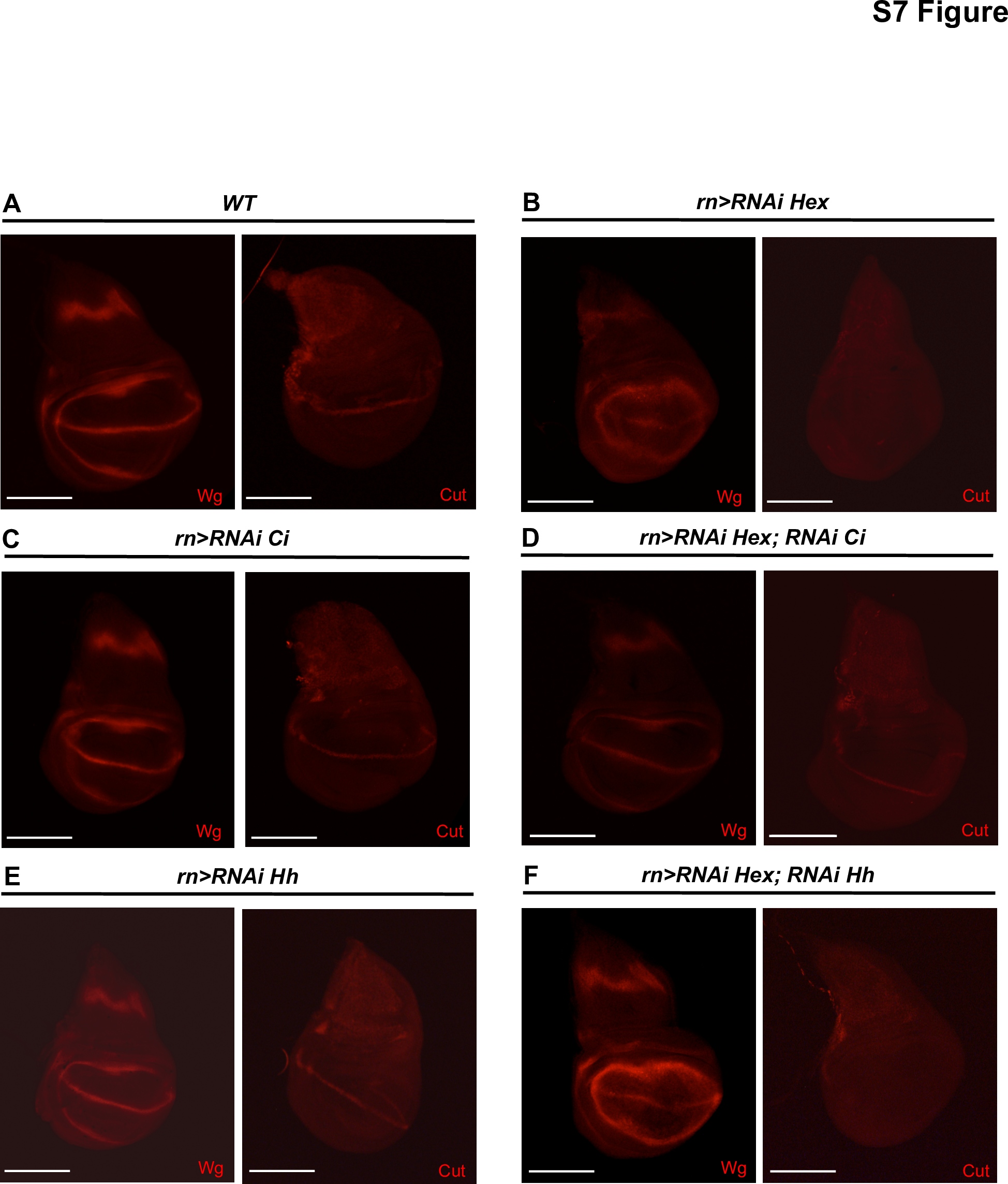

Supplement: S7 Fig — Immuno-staining of Wg and Cut in WT (A), rn-Gal4>RNAi Hexim (B), rn-Gal4>RNAi Ci (C), rn-Gal4>RNAi Hexim; RNAi Ci (D), rn-Gal4>RNAi Hh (E) and rn-Gal4>RNAi Hexim; RNAi Hh (F). (TIF) [file pone.0155438.s007.tif]

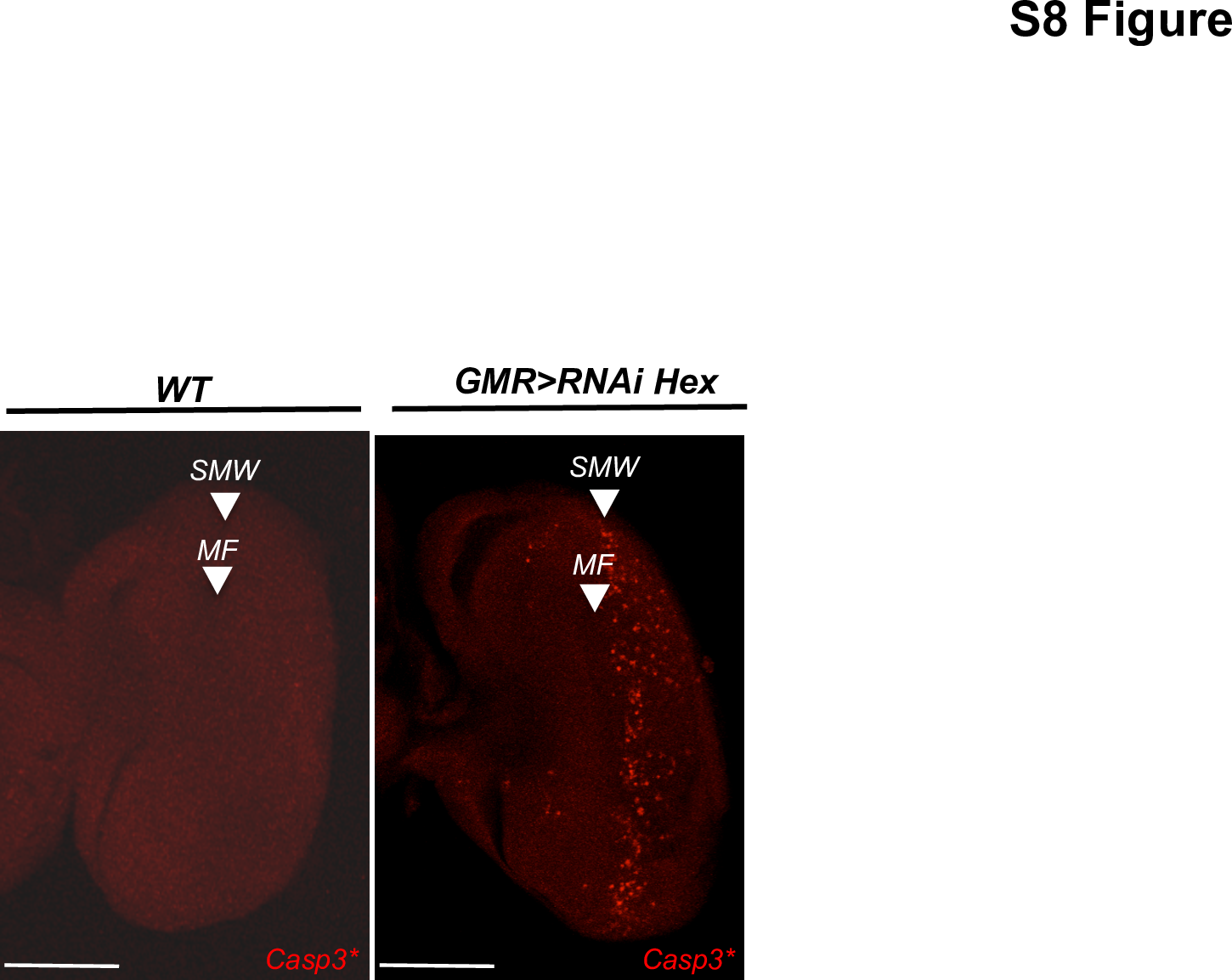

Supplement: S8 Fig — Immuno-localization at ealy L3 stage of Casp3* in WT and GMR-Gal4>RNAi Hexim eye discs. MF: Morphogenic Furrow. SMW: Secondary Mitotic Wave. (TIF) [file pone.0155438.s008.tif]
