# Supplementary material for: Functional Interaction between HEXIM and Hedgehog Signaling during Drosophila Wing Development
Source: PLoS One. 2016 May 13;11(5):e0155438. doi: 10.1371/journal.pone.0155438 (PMC4866710; doi:10.1371/journal.pone.0155438)
Supplement: S1 Table — (PDF) [file pone.0155438.s009.pdf]

S1 Table. **Phenotype of Hh or HEXIM RNAi mutants.**

Genotype[e]	Phenotype	Results[a]
rn>RNAi Hh	anterior crossvein size/ wing size	54,4 % (+/- 10,4)[b]
	L3-L4 area / wing size	89 % (+/- 3,1)[c]
so>RNAi Hex	Rought eyes	100 %
	Small eyes	61,3 % (+/- 4,8) (male); 75,1 % (+/- 4,8) (female)[d]

[a] Phenotype penetrance is 100% in all cases and they are statistically significant.

[b] Reduction of the anterior crossvein/total wing size ratio (expressed in % of WT); +/-, standard deviation.

[c] Reduction of the L3-L4 area/total wing size ratio (expressed in % of WT).

[d] Eye size is expressed as a % of WT eyes. This phenotype displays a sexual dimorphism.

[e] *rn*: *rotund* ; *so*: *sine-oculis*.
