# Supplementary material for: Functional Interaction between HEXIM and Hedgehog Signaling during Drosophila Wing Development
Source: PLoS One. 2016 May 13;11(5):e0155438. doi: 10.1371/journal.pone.0155438 (PMC4866710; doi:10.1371/journal.pone.0155438)
Supplement: S2 Table — (DOC) [file pone.0155438.s010.doc]

**S2 Table:** List of differentially expressed genes in *GMR-Gal4>RNAi Hex* adult heads compared to wild type.

| **Log Fold-Change^1^** | **adjusted P-Value^2^** | **ID** | **Gene** | **FlyBase ID** | **Gene Ontology Biological Process^3^** |
| --- | --- | --- | --- | --- | --- |
| 4,93 | 0,0190 | CG10146-RA | attacin | FBgn0012042 | immune response |
| 3,77 | 0,0001 | CG2759-RA | white | FBgn0003996 | eye pigment biosynthetic process |
| 3,55 | 0,0144 | CG18372-RA | attacin | FBgn0012042 / FBgn0041581 | defense response |
| 3,51 | 0,0334 | CG1373-RA | Cecropin | FBgn0000279 | immune response |
| 3,29 | 0,0001 | CG9150-RA | CG9150 | FBgn0031775 | metabolic process |
| 2,90 | 0,0025 | CG11997-RA | CG11997 | FBgn0037662 | --- |
| 2,54 | 0,0032 | CT32157 | CG30080 | FBgn0050080 | regulation of transcription, DNA-dependent |
| 2,49 | 0,0083 | CG33468-RA | CG33468 | FBgn0053468 | --- |
| 2,16 | 0,0288 | CG30080-RA | CG30080 | FBgn0050080 | regulation of transcription, DNA-dependent |
| 1,95 | 0,0120 | CG10924-RA | CG10924 | FBgn0034356 | gluconeogenesis |
| 1,95 | 0,0293 | CG1151-RA | Osiris | FBgn0027527 | --- |
| 1,87 | 0,0070 | CG32553-RA | CG32553 | FBgn0052553 | --- |
| 1,84 | 0,0014 | CG4466-RA | heat shock protein hsp27 | FBgn0001226 | response to stress |
| 1,70 | 0,0032 | Transposon.11 | gag-int-pol | FBgn0013437 | DNA integration |
| 1,64 | 0,0005 | CG4463-RA | Protein 23 | FBgn0001224 | response to hypoxia |
| 1,62 | 0,0334 | CG14567-RA | CG14567 | FBgn0037126 | --- |
| 1,62 | 0,0282 | CG31704-RA | CG31704 | FBgn0051704 | proteolysis |
| 1,61 | 0,0161 | CG7906-RA | CG7906 | FBgn0036417 | --- |
| 1,56 | 0,0275 | CG11709-RA | semmelweis | FBgn0030310 | defense response |
| 1,48 | 0,0014 | CG16928-RA | meiotic recombination 11 | FBgn0020270 | telomere maintenance |
| 1,43 | 0,0153 | CG8620-RA | CG8620 | FBgn0040837 | --- |
| 1,34 | 0,0032 | CG5697-RA | CG5697 | FBgn0038846 | --- |
| 1,30 | 0,0064 | CG10102-RA | CG12505 / CG10102 | FBgn0033926 / FBgn0033927 | --- |
| 1,28 | 0,0170 | CG18466-RB | NAD-dependent methylene tetrahydrofolate dehydrogenase | FBgn0010222 | carbohydrate metabolic process |
| 1,20 | 0,0187 | CG17285-RA | Protein-1 | FBgn0000639 | transport |
| 1,18 | 0,0222 | CG2772-RA | CG2772 | FBgn0031533 | lipid metabolic process |
| 1,16 | 0,0246 | CG5359-RA | CG5359 | FBgn0037773 | --- |
| 1,13 | 0,0119 | CG14620-RA | touch insensitive larva B | FBgn0014395 | sensory perception of sound |
| 1,12 | 0,0066 | CG2849-RB | anon-fast-evolving-1H4 | FBgn0015286 | signal transduction |
| 1,06 | 0,0246 | CG18559-RA | Cyp309a2 | FBgn0041337 | oxidation reduction |
| 1,06 | 0,0064 | CG2064-RA | CG2064 | FBgn0033205 | metabolic process |
| 1,05 | 0,0484 | CT33033 | --- | --- | --- |
| 1,05 | 0,0070 | CG15117-RA | CG15117 | FBgn0034417 | carbohydrate metabolic process |
| 0,98 | 0,0224 | CG32244-RB | spz2 | FBgn0260984 | --- |
| 0,97 | 0,0024 | CG5224-RA | CG5224 | FBgn0034354 | --- |
| 0,96 | 0,0120 | CG9325-RB | hu-li tai shao | FBgn0004873 | meiotic spindle organization |
| 0,91 | 0,0413 | CG1944-RA | Cyp4p2 | FBgn0033395 | oxidation reduction |
| 0,87 | 0,0246 | CG8213-RA | CG8213 | FBgn0033359 | proteolysis |
| 0,84 | 0,0032 | CG10383-RA | CG10383 | FBgn0032699 | --- |
| 0,83 | 0,0238 | CG13835-RA | CG13834 | FBgn0085404 | ubiquitin-dependent protein catabolic process |
| 0,81 | 0,0033 | CG8981-RA | --- | --- | --- |
| 0,78 | 0,0246 | CG10961-RA | TNF-receptor-associated factor 2 | FBgn0026318 | defense response |
| 0,77 | 0,0065 | CG8768-RA | CG8768 | FBgn0033769 | metabolic process |
| 0,73 | 0,0147 | Dm.3R.41869.0 | CG17816 | FBgn0037525 | --- |
| 0,72 | 0,0120 | CG13941-RA | CG13941 | FBgn0033928 | --- |
| 0,71 | 0,0307 | CG7763-RA | CG7763 | FBgn0040503 | --- |
| 0,70 | 0,0144 | CG15281-RA | CG30287 / --- | FBgn0050287 / FBgn0085195 | proteolysis |
| 0,70 | 0,0065 | CG6830-RA | CG6830 | FBgn0037934 | --- |
| 0,69 | 0,0233 | CG11671-RA | CG11671 | FBgn0037562 | --- |
| 0,69 | 0,0162 | Transposon.33 | --- | --- | --- |
| 0,67 | 0,0120 | CG12116-RA | CG12116 / --- | FBgn0030041 / FBgn0261193 | metabolic process |
| 0,67 | 0,0120 | CG13299-RA | CG13299 | FBgn0035694 | --- |
| 0,67 | 0,0154 | CG14529-RA | CG14529 | FBgn0039609 | proteolysis |
| 0,65 | 0,0246 | CG17224-RA | CG17224 | FBgn0031489 | nucleoside metabolic process |
| 0,62 | 0,0153 | LP09838 | --- | --- | --- |
| 0,60 | 0,0419 | CG12716-RA | CG12716 | FBgn0030439 | --- |
| 0,60 | 0,0061 | CG31780-RB | CG18477 / CG31780 | FBgn0028864 / FBgn0051780 | proteolysis |
| 0,60 | 0,0036 | CG33209-RA | comm3 | FBgn0259236 | salivary gland cell autophagic cell death |
| 0,59 | 0,0153 | CG8193-RA | CG8193 | FBgn0033367 | transport |
| 0,58 | 0,0487 | CG4859-RA | Matrix metalloproteinase 1 | FBgn0035049 | instar larval development |
| 0,57 | 0,0246 | CG2647-RA | period | FBgn0003068 | negative regulation of transcription from RNA polymerase II promoter |
| 0,56 | 0,0361 | CG13627-RA | CG13627 | FBgn0039217 | --- |
| 0,55 | 0,0226 | CG7381-RC | CG7381 | FBgn0038098 | --- |
| 0,55 | 0,0453 | CG12268-RA | CG12268 | FBgn0039131 | metabolic process |
| 0,54 | 0,0487 | CG30280-RA | CG30280 | FBgn0050280 | signal transduction |
| 0,54 | 0,0116 | CG10337-RA | CG10337 | FBgn0032805 | --- |
| 0,53 | 0,0290 | CG14495-RA | CG14495 | FBgn0034293 | --- |
| 0,50 | 0,0144 | CG16901-RA | RNA-binding protein 3 | FBgn0086897 | nuclear-transcribed mRNA catabolic process, nonsense-mediated decay |
| 0,50 | 0,0290 | CG10017-RA | CG10017 | FBgn0085369 | regulation of transcription, DNA-dependent |
| 0,50 | 0,0352 | LD11162 | CG42384 / CG42385 / CG42386 / CG42387 | FBgn0259730 / FBgn0259731 / FBgn0259732 / FBgn0259733 | --- |
| 0,49 | 0,0155 | CG40100-RA | CG30022 / CG40100 | FBgn0050022 / FBgn0058100 | --- |
| 0,49 | 0,0458 | CG7122-RB | RhoGAP16F | FBgn0030893 | signal transduction |
| 0,48 | 0,0238 | CG31220-RA | CG31220 | FBgn0051220 | proteolysis |
| 0,48 | 0,0127 | CG15484-RA | CG15484 | FBgn0032452 | --- |
| 0,48 | 0,0162 | CG15154-RA | Suppressor of cytokine signaling at 36E | FBgn0041184 | intracellular signaling cascade |
| 0,46 | 0,0246 | CG17264-RA | CG17264 | FBgn0031490 | --- |
| 0,46 | 0,0458 | CG1630-RA | Inositol 1,4,5-triphosphate kinase 2 | FBgn0085388 | inositol and derivative phosphorylation |
| 0,44 | 0,0125 | CG3831-RA | CG3831 / --- | FBgn0034804 / FBgn0086525 | --- |
| 0,44 | 0,0120 | CG17544-RA | CG17544 | FBgn0032775 | fatty acid metabolic process |
| 0,44 | 0,0163 | CG13046-RA | CG13046 | FBgn0036595 | --- |
| 0,44 | 0,0238 | CG17611-RA | eIF6 | FBgn0034915 | translation |
| 0,44 | 0,0144 | CG3376-RB | CG3376 | FBgn0034997 | sphingomyelin catabolic process |
| 0,43 | 0,0144 | CT33773 | --- | --- | --- |
| 0,43 | 0,0280 | CG18314-RA | DopEcR | FBgn0035538 | G-protein coupled receptor protein signaling pathway |
| 0,41 | 0,0402 | CG13610-RA | Organic cation transporter 2 | FBgn0086365 | transport |
| 0,40 | 0,0246 | CG9273-RA | CG9273 | FBgn0032906 | DNA-dependent DNA replication |
| 0,40 | 0,0352 | CG10916-RA | CG10916 | FBgn0034312 | --- |
| 0,37 | 0,0246 | CG32313-RA | CG32313 | FBgn0052313 | --- |
| 0,35 | 0,0246 | CG5921-RA | CG5921 | FBgn0029835 | --- |
| 0,35 | 0,0307 | CG13791-RA | CG13791 | FBgn0031923 | --- |
| 0,33 | 0,0294 | CG8865-RB | Ral guanine nucleotide exchange factor 2 | FBgn0026376 | signal transduction |
| 0,33 | 0,0377 | CG3168-RC | CG3168 | FBgn0029896 | DNA topological change |
| 0,32 | 0,0370 | CG5638-RA | rhodopsin | FBgn0036260 | signal transduction |
| 0,32 | 0,0383 | CG11898-RA | CG11898 | FBgn0039645 | transport |
| 0,32 | 0,0435 | CG3568-RA | CG3568 | FBgn0029710 | --- |
| 0,30 | 0,0280 | CG3448-RB | CG3448 | FBgn0035996 | --- |
| -0,28 | 0,0487 | Dm.X.55357.0 | --- | --- | --- |
| -0,29 | 0,0478 | CG6843-RA | CG6843 | FBgn0036827 | --- |
| -0,29 | 0,0487 | CG8862-RA | CG8862 | FBgn0033690 | DNA-dependent DNA replication |
| -0,30 | 0,0396 | CG13473-RA | CG13473 | FBgn0036442 | cell redox homeostasis |
| -0,31 | 0,0251 | CG8108-RA | CG8108 | FBgn0027567 | --- |
| -0,31 | 0,0397 | CG9790-RA | Cyclin-dependent kinase subunit 85A | FBgn0037613 | cell cycle |
| -0,32 | 0,0490 | CG8573-RB | suppressor of hairy-wing | FBgn0003567 | transcription |
| -0,32 | 0,0490 | CG11711-RC | Mob2 | FBgn0259481 | cell morphogenesis |
| -0,32 | 0,0487 | CG3399-RC | cappuccino | FBgn0000256 | multicellular organismal development |
| -0,32 | 0,0377 | CG16885-RA | CG16885 | FBgn0032538 | --- |
| -0,33 | 0,0355 | CG6097-RA | Drosophila Protein O-mannosyltransferase 1 | FBgn0003292 | protein amino acid O-linked glycosylation |
| -0,33 | 0,0275 | CG4184-RA | Mediator complex subunit 15 | FBgn0027592 | transcription |
| -0,33 | 0,0315 | CG7928-RA | CG7928 | FBgn0039740 | --- |
| -0,34 | 0,0466 | CG6258-RA | DNA replication factor, 38k subunit | FBgn0028700 | DNA replication |
| -0,35 | 0,0366 | CG7289-RA | CG7289 | FBgn0031379 | --- |
| -0,35 | 0,0307 | CG9029-RA | CG9029 | FBgn0031746 | defense response |
| -0,35 | 0,0484 | CG8043-RA | CG8043 | FBgn0037610 | glycine catabolic process |
| -0,36 | 0,0246 | CG14728-RA | shadow | FBgn0003312 | ecdysone biosynthetic process |
| -0,36 | 0,0214 | CG13855-RA | CG13855 | FBgn0038960 | --- |
| -0,36 | 0,0170 | S.CX000354 | --- | --- | --- |
| -0,36 | 0,0487 | CG7221-RA | Wwox | FBgn0031972 | metabolic process |
| -0,37 | 0,0282 | CG5792-RA | CG5792 | FBgn0032455 | --- |
| -0,37 | 0,0170 | CG7227-RA | CG7227 | FBgn0031970 | defense response |
| -0,38 | 0,0181 | CG2248-RA | Rac1 | FBgn0010333 | phagocytosis triggered by activation of immune response cell surface activating receptor |
| -0,39 | 0,0275 | CG3998-RA | Zinc finger protein 30C | FBgn0022720 | neuron development |
| -0,40 | 0,0307 | CG7714-RA | CG7714 | FBgn0038645 | chitin metabolic process |
| -0,41 | 0,0190 | CG7068-RA | Thiolester containing protein III | FBgn0041181 | phagocytosis, engulfment |
| -0,42 | 0,0153 | CG6191-RA | CG6191 | FBgn0027581 | phagocytosis, engulfment |
| -0,42 | 0,0438 | Transposon.44 | --- | --- | --- |
| -0,42 | 0,0125 | CG17931-RB | CG17931 | FBgn0038421 | --- |
| -0,43 | 0,0304 | CG12189-RA | Rev1 | FBgn0035150 | DNA repair |
| -0,43 | 0,0165 | CG4596-RA | CG4596 | FBgn0037849 | --- |
| -0,43 | 0,0246 | CG3642-RA | Clipper | FBgn0015621 | mRNA polyadenylation |
| -0,44 | 0,0144 | CG8599-RA | Suppressor of variegation 3-7 | FBgn0003598 | --- |
| -0,44 | 0,0242 | CG32922-RA | CG14681 | FBgn0037820 | histidine catabolic process |
| -0,44 | 0,0155 | CG3524-RA | v(2)k05816 | FBgn0042627 | metabolic process |
| -0,44 | 0,0306 | S.C3L000477 | --- | --- | --- |
| -0,45 | 0,0170 | CG11170-RB | CG11170 | FBgn0034705 | --- |
| -0,45 | 0,0344 | CG4200-RA | phospholipase Cgamma | FBgn0003416 | lipid metabolic process |
| -0,45 | 0,0153 | CG3386-RA | CG3386 | FBgn0035152 | --- |
| -0,45 | 0,0155 | CG8977-RA | predicted gene Y | FBgn0015019 | protein folding |
| -0,46 | 0,0072 | CG1919-RA | CG1919 | FBgn0035281 | --- |
| -0,48 | 0,0392 | CG10327-RC | TBPH | FBgn0025790 | neuromuscular junction development |
| -0,48 | 0,0266 | CG13935-RA | CG13935 | FBgn0035280 | --- |
| -0,48 | 0,0258 | CG33154-RB | CG13175 / CG33964 | FBgn0033693 / FBgn0053964 | --- |
| -0,49 | 0,0232 | CG13065-RA | CG13065 | FBgn0036590 | --- |
| -0,50 | 0,0451 | CG11345-RA | CG11345 | FBgn0035546 | --- |
| -0,50 | 0,0120 | CG3552-RA | CG3552 | FBgn0035999 | --- |
| -0,50 | 0,0487 | CG9476-RA | alpha-Tubulin | FBgn0003886 | microtubule-based process |
| -0,50 | 0,0170 | CG13081-RA | CG13081 | FBgn0032804 | --- |
| -0,51 | 0,0065 | CG31332-RC | --- | --- | cytoskeleton organization |
| -0,54 | 0,0275 | CG32810-RB | CG32810 | FBgn0025394 | ion transport |
| -0,54 | 0,0458 | CG14302-RA | CG14302 | FBgn0038647 | --- |
| -0,54 | 0,0064 | CG18542-RA | CG18542 | FBgn0037731 | --- |
| -0,55 | 0,0397 | CG12749-RA | Heterogeneous nuclear ribonucleoprotein at 85CD | FBgn0004237 | regulation of alternative nuclear mRNA splicing, via spliceosome |
| -0,55 | 0,0173 | CG7402-RA | CG7402 | FBgn0036768 | metabolic process |
| -0,58 | 0,0438 | CG13056-RA | CG13056 | FBgn0040794 | --- |
| -0,60 | 0,0170 | CG6754-RB | lethal (3) 67BDp | FBgn0086349 | telomere maintenance |
| -0,60 | 0,0334 | CG4322-RA | moody | FBgn0025631 | signal transduction |
| -0,61 | 0,0382 | CG11205-RA | CG18853 / photolyase | FBgn0003082 / FBgn0042173 | DNA repair |
| -0,61 | 0,0036 | CG17958-RA | serendipity delta | FBgn0003512 | transcription |
| -0,63 | 0,0033 | CG11680-RB | no action potential | FBgn0002774 | dosage compensation |
| -0,64 | 0,0282 | CG11409-RB | CG11409 / CG11409 | FBgn0024366 / FBgn0084757 | --- |
| -0,64 | 0,0064 | CG31759-RB | CG31759 | FBgn0051759 | methylation |
| -0,64 | 0,0248 | CG32146-RA | Dally-like | FBgn0041604 | smoothened signaling pathway |
| -0,64 | 0,0334 | CG8568-RA | CG8568 | FBgn0030841 | --- |
| -0,64 | 0,0258 | CG6646-RA | DJ-1alpha | FBgn0033885 | response to oxidative stress |
| -0,65 | 0,0292 | CG5660-RA | CG5660 | FBgn0035942 | translation |
| -0,66 | 0,0238 | CG2044-RA | CG11018 / cuticle p. IV | FBgn0002535 / FBgn0034464 | --- |
| -0,66 | 0,0366 | CG10513-RA | CG10513 | FBgn0039311 | --- |
| -0,68 | 0,0119 | CG10638-RA | CG10638 | FBgn0036290 | cellular aldehyde metabolic process |
| -0,68 | 0,0348 | AFFX-Dm-Gpdh-3 | --- | --- | --- |
| -0,68 | 0,0246 | CG32072-RA | CG32072 | FBgn0052072 | very-long-chain fatty acid metabolic process |
| -0,69 | 0,0281 | CG9042-RB | 5' gene | FBgn0001128 | carbohydrate metabolic process |
| -0,69 | 0,0226 | CG15106-RA | Juvenile hormone epoxide hydrolase 3 | FBgn0034406 | juvenile hormone catabolic process |
| -0,70 | 0,0056 | CG13067-RA | CG13067 | FBgn0036589 | --- |
| -0,71 | 0,0119 | CG7077-RA | CG7077 / CG7077 / CG7077 / CG7077 | FBgn0038946 / FBgn0086919 / FBgn0086951 / FBgn0086952 | metabolic process |
| -0,74 | 0,0021 | CG15080-RA | CG15080 | FBgn0034391 | --- |
| -0,74 | 0,0191 | CG8587-RA | Cyp301a1 | FBgn0033753 | oxidation reduction |
| -0,80 | 0,0033 | CG15582-RA | Odorant-binding protein 83d | FBgn0046878 | transport |
| -0,80 | 0,0064 | CG15739-RA | CG15739 | FBgn0030347 | metabolic process |
| -0,81 | 0,0068 | CG32918-RA | CG15891 / CG15892 | FBgn0029859 / FBgn0029860 | --- |
| -0,84 | 0,0144 | CG40485-RB | CG40485 | FBgn0069973 | metabolic process |
| -0,85 | 0,0048 | CG6781-RA | sepia | FBgn0086348 | eye pigment biosynthetic process |
| -0,95 | 0,0021 | CG11066-RB | scarface | FBgn0033033 | proteolysis |
| -0,95 | 0,0064 | CG11822-RA | Dbeta3 | FBgn0031261 | transport |
| -0,96 | 0,0361 | CG5494-RA | CG5494 | FBgn0038819 | --- |
| -1,03 | 0,0315 | CG6472-RA | CG6472 | FBgn0034166 | lipid metabolic process |
| -1,03 | 0,0163 | CG1851-RA | Ady43A | FBgn0026602 | purine ribonucleoside salvage |
| -1,08 | 0,0032 | CG13101-RA | CG13101 | FBgn0032084 | --- |
| -1,09 | 0,0161 | CT34163 | gp210 | FBgn0033039 | learning or memory |
| -1,18 | 0,0003 | CG12408-RA | TpnC4 | FBgn0033027 | ciliary or flagellar motility |
| -1,21 | 0,0221 | CG8510-RA | CG8510 | FBgn0033729 | --- |
| -1,23 | 0,0120 | CG10407-RA | CG10407 | FBgn0038395 | --- |
| -1,37 | 0,0327 | CG7361-RA | Rieske iron-sulfur protein | FBgn0021906 | mitochondrial electron transport, ubiquinol to cytochrome c |
| -1,37 | 0,0125 | CG30450-RA | Odorant-binding protein 56f | FBgn0043533 | transport |
| -1,40 | 0,0061 | CG4125-RA | irregular chiasm C-roughest | FBgn0003285 | compound eye morphogenesis |
| -1,54 | 0,0392 | CG1461-RA | CG1461 | FBgn0030558 | cellular amino acid and derivative metabolic process |
| -1,58 | 0,0025 | Transposon.47 | --- | --- | --- |
| -1,72 | 0,0001 | CG1705-RA | methoprene-tolerant | FBgn0002723 | transcription |
| -2,85 | 0,0066 | CG5279-RA | Rhodopsin5 | FBgn0014019 | signal transduction |

^1^ Log Fold-Change : Logarithm of the ratio between the signal intensity of the mutant over wild-type. Since it is expressed in log base 2, a Log Fold-Change value of 1 corresponds to a transcriptional induction of two folds. In red are the genes which are overexpressed, in green the ones which are down-expressed.

^2^ Adjusted P-value : P-value corrected for multiple testing.

^3^ --- : indicate genes encoding proteins with unknown function.
